# Leaf gas films enhance metabolic responses to submergence in *Cynodon dactylon*

**DOI:** 10.3389/fpls.2026.1786650

**Published:** 2026-06-02

**Authors:** Feixue Xia, Liangzhuang Geng, Feng Lin, Linsha Chen, Fusen Huang, Wanting Zhang, Xiaoping Liao, Junchao Hong, Kai Wang, Bo Zeng, Xiaoping Zhang, Qiaoli Ayi

**Affiliations:** 1 Key Laboratory of Eco-environments in Three Gorges Reservoir Region (Ministry of Education), School of Life Sciences, Southwest University, Chongqing, China; 2 Chongqing Key Laboratory of Plant Ecology and Resources in Three Gorges Reservoir Region, School of Life Sciences, Southwest University, Chongqing, China

**Keywords:** aerobic metabolism, central carbon metabolism, *Cynodon dactylon*, flooding tolerance, floodplain ecosystem adaptation, riparian plant resilience

## Abstract

Riparian plants frequently experience flooding, which imposes oxygen limitation and disrupts carbohydrate metabolism, threatening survival and post-flood recovery. Certain species, including *Cynodon dactylon*, form leaf gas films—thin layers of air retained on hydrophobic surfaces—that may alleviate stress hypoxia. However, their effects on central carbon metabolism remain poorly understood. Here, we investigated the role of leaf gas films in the submergence tolerance of *C. dactylon* by integrating physiological measurements, non-structural carbohydrate analysis, and targeted metabolomics. Plants were subjected to complete submergence with or without gas film retention, and growth, stomatal behavior, oxygen availability, carbohydrate consumption, and metabolite profiles were monitored. Gas films formed rapidly upon submergence, enhancing stomatal opening and maintaining higher oxygen partial pressures near the leaf surface. Submerged plants with intact gas films retained more green leaves, exhibited faster stem elongation, and preserved on non-structural carbohydrates in their leaves compared with plants lacking gas films. Metabolomic analyses revealed that gas films sustained flux through the tricarboxylic acid cycle and the pentose phosphate pathway. This supported uracil biosynthesis and aerobic energy metabolism. In contrast, plants without gas films shifted towards fermentative and secondary metabolic pathways. Gas films persisted for approximately seven days, providing a transient but critical window for aerobic metabolism under flooding conditions. These findings demonstrate that leaf gas films function as an early-phase adaptive mechanism, that promotes carbohydrate homeostasis and energy balance during submergence. By sustaining aerobic respiration and growth, gas films likely contribute to individual survival, competitive advantage, and the ecological resilience of riparian communities under fluctuating water levels.

## 1 Introduction

Riparian zones are dynamic interfaces between terrestrial and aquatic ecosystems that play essential roles in maintaining water quality, regulating hydrological processes and supporting biodiversity (Bren, 1993; Allan et al., 2008; Urbanič et al., 2022). Vegetation is a cornerstone of riparian function: it stabilizes soils, intercepts nutrients and pollutants, moderates local microclimate, and supplies habitat and resources for a wide range of organisms (Macfarlane et al., 2017; Riis et al., 2020). Consequently, the composition and resilience of riparian vegetation communities are tightly linked to ecosystem health and functioning.

Flooding is a defining disturbance in riparian landscapes. Periodic inundation arises from both natural hydrological fluctuations and anthropogenic drivers such as dam regulation and reservoir operation (Aguiar et al., 2018; Su et al., 2020). In the context of global environmental change, intensifying rainfall extremes and increasing flood frequency are predicted to exacerbate these hydrological disturbances worldwide (Hirabayashi et al., 2013; IPCC, 2014). Such pressures are expected to have profound ecological consequences, including altered community composition, shifts in vegetation zonation and reduced ecosystem stability in riparian environments.

For plants, submergence imposes a unique suite of challenges distinct from those encountered under other abiotic stresses. In contrast to permanently submerged macrophytes that are adaptive to aquatic life, most riparian plants are primarily adapted to aerial environments and rely on stomatal gas exchange (Overdieck et al., 2013; Garssen et al., 2015) (Overdieck et al., 2013; Garssen et al., 2015). When suddenly submerged, these plants experience sharp declines in light penetration, CO_2_ availability and oxygen diffusion (Armstrong, 1979; Sand-Jensen and Krause-Jensen, 1997; Andersen and Pedersen, 2002). Photosynthetic carbon gain is severely inhibited, while oxygen deprivation triggers fermentative metabolism that accelerates carbohydrate depletion (Gibbs and Greenway, 2003; Nurrahma et al., 2021). Together, these constraints create a major bottleneck for plant survival under flooding.

Plants have evolved various strategies to cope with submergence, including aerenchyma formation, shoot elongation and metabolic adjustment (Ayi et al., 2016). Well-studied examples include the development of aerenchyma, enhanced shoot elongation, or shifts towards more efficient anaerobic metabolism. More recently, attention has turned to the phenomenon of leaf gas films. Certain species can retain a thin layer of air along the hydrophobic surfaces of their leaves when submerged, forming so-called gas films. Analogous to the plastron respiration of aquatic insects (Thorpe and Crisp, 1946; 1947a, b; 1949), these films maintain stomatal contact with a gaseous environment, enhancing the diffusion of O_2_ and CO_2_ across the leaf surface (Colmer and Pedersen, 2008a; Pedersen et al., 2009; Winkel et al., 2011). Experimental studies have shown that gas films can substantially increase underwater photosynthetic rates and reduce oxygen deficiency damage (Verboven et al., 2014; Konnerup and Pedersen, 2017; Kurokawa et al., 2018). However, their persistence varies among species and their broader ecological significance remains debated (Winkel et al., 2016; Konnerup et al., 2017).

Despite growing interest in gas films, most studies have focused on their immediate role in facilitating gas exchange. In particular, it remains unclear whether gas films affect central carbon metabolism (CCM), which integrates energy production and biosynthesis. Under submergence, restricted oxygen availability typically constrains aerobic metabolism and accelerates carbohydrate consumption (Gibbs and Greenway, 2003; Pramanik et al., 2016). If gas films improve oxygen supply, they may not only delay carbohydrate depletion but also enable finer regulation of CCM, thus supporting energy homeostasis and enhancing flood tolerance. Yet, this mechanistic link remains largely hypothetical and has rarely been tested in riparian species.

*Cyondon dactylon* (Bermudagrass) is a perennial grass widely distributed in riparian zones and drawdown areas. It is known for its strong flood tolerance and is frequently used in ecological restoration of reservoir shorelines. Despite its ecological importance, the interaction between gas film formation and metabolic regulation in this species remains poorly understood.

Here, we investigated whether gas films in *C. dactylon* influence oxygen availability and metabolic responses under complete submergence. Specifically, we addressed: (1) Does *C. dactylon* form gas films under complete submergence, and how do these films contribute to its flood tolerance?

(2) Are leaf gas films associated with changes in carbohydrate dynamics and CCM-related pathways??

To explore these questions, we conducted a comprehensive analysis of gas film formation, plant growth, non-structural carbohydrate (NSC) dynamics and CCM responses in *C. dactylon* under complete submergence. By integrating structural and metabolic perspectives, our study seeks to clarify the functional significance of gas films in flood tolerance and to provide new insights into their ecological role in maintaining riparian vegetation performance and community resilience under increasing hydrological disturbance.

## 2 Materials and methods

### 2.1 Plant material and growth conditions

Specimens of *C. dactylon* were randomly sampled from populations in the experimental garden of the Key Laboratory of Eco-environments in Three Gorges Reservoir Region in Southwest University, Chongqing, China. These plants originated from the same site at the bank of Jialing River in Beibei (29° 50′N, 106° 26′E).

Plants used in this study were cultivated from cuttings of unbranched shoots, approximately 35 cm in length, obtained from donor plants. Each cutting was grown individually in pots (Ø = 15 cm, H = 15 cm) filled with a soil mixture of 40% clay, 40% humus soil and 20% sand, and two stem nodes were buried in the soil for rooting. The plants were cultivated outdoors under ambient conditions, i.e., daytime temperature 15-20 °C, relative humidity 75-85%, light (PAR) 600-800 µmol m^-2^ s^-1^, and provided with ample water (~80-90% of soil water-holding capacity) using tap water. After one month, the plants had grown well and developed approximately 23–27 leaves. Plants were randomly assigned to treatments, and all measurements were performed using independent biological replicates.

### 2.2 Experimental design and treatments

Plants, approximately 35 cm in length, were assigned to one of five groups (15 pots per group): one baseline group and four experimental treatments, (1) unsubmerged with gas film retained (W−T−), (2) unsubmerged with gas film removed (W−T+), (3) fully submerged with gas film retained (W+T−), and (4) fully submerged with gas film removed (W+T+). Plants were in baseline group definition as plants harvested before treatment initiation to determine the initial physiological and biochemical status. Gas films were removed by gently brushing the adaxial and abaxial leaf surfaces with cotton soaked in 0.05% Triton X-100.

Four treatments were applied in a fully randomized design using selected plants. Submerged plants were placed in a water-filled concrete reservoir (length × width × depth:2.3 × 1.9 × 3 m), with the tops of the plants positioned 2 m below the water surface. Potted plants were suspended at predetermined water depths. During the submergence period, the physical-chemical status of the water [light, dissolved oxygen (DO), pH, and temperature] was kept constant. The water was air-saturated by daily aeration through an air pipe with vent holes installed at the reservoir bottom, ensuring an adequate and uniform oxygen supply, and kept the DO around 100% air saturation. Water temperature was maintained around 20 ± 2°C using an electric heating system installed at the bottom. During the submergence treatment, plants were supplied with artificial illumination from LED tubes, maintaining a light intensity of 250–300 μmol m^-^² s^-^¹ (PAR). The reservoir was covered with black sun-shading nets to eliminate light. The pH of the water was maintained at approximately 7.0–7.5, corresponding to the natural pH range of local tap water. DO concentration, temperature, and pH in the water column were measured twice daily (morning and evening) using a multi-parameter water-quality analyzer (Hydrolab DS5, Hach, United States). No significant differences in these parameters were detected during the experiment. The unsubmerged treatment was conducted in darkness at the same temperature as the submergence treatments. Unsubmerged plants were watered normally to maintain soil water at field capacity.

### 2.3 Gas film formation and stomatal behaviors

Gas film formation on leaf surfaces was assessed through contact angle measurements and oxygen microelectrode profiling. The presence of gas films relies on a non-wetting interface between water and the leaf surface, which is determined by hydrophobic or superhydrophobic surface structures. To evaluate surface wettability, the contact angle of a water droplet on the leaf surface was measured, with surfaces categorized as hydrophilic (<90°), hydrophobic (90–150°), or superhydrophobic (>150°) (Nosonovsky and Bhushan, 2005; 2007). For each plant, the first fully expanded leaf below the stem apex was selected. A 5 µL droplet of deionized water was gently placed at the center of the lamina (avoiding major veins) from a height of 2–3 cm using a micropipette. Contact angles were recorded using a horizontal microscope (CEWEI, China) and subsequently analyzed with ImageJ software. Measurements were conducted on both adaxial and abaxial surfaces under controlled laboratory conditions (20 °C, 64% relative humidity).

To investigate oxygen dynamics near the leaf surface, oxygen concentration profiles were measured using a Clark-type oxygen microelectrode (tip diameter 100 µm; OX100, Unisense, Denmark) mounted on a motor-controlled micromanipulator. The electrode was advanced from the bulk water toward the leaf surface at a rate of 3 µm s^-^¹, with signals amplified by a picoammeter and subsequently converted to oxygen concentrations. Measurements were first recorded within the gas film adjacent to the leaf surface. Subsequently, the microelectrode was carefully inserted into the leaf tissue to determine internal oxygen concentration. This stepwise profiling approach allowed us to distinguish between oxygen availability in the gas film and oxygen levels within leaf tissues. Measurements were conducted in the central lamina region, avoiding major veins, under dark conditions. To assess the influence of cuticular wax removal, the adaxial surface of leaves was gently brushed with 0.05% (v/v) Triton X-100, rinsed three times with ultrapure water, and then re-profiled. The same procedure was also applied to the abaxial surface. Triton X-100 solution was used to remove leaf gas films. This concentration is widely applied to reduce surface tension and disrupt hydrophobic interfaces while minimizing damage to leaf tissues. Potential effects on leaf surface properties are acknowledged; however, this treatment is effective for isolating the functional role of gas films.

Beginning from the onset of submergence, three plants were sampled each day. For every leaf, contact angle measurements were taken prior to microelectrode profiling. Gas film thickness was then estimated from oxygen gradients across the diffusion boundary layer (DBL), with a thickness < 2 µm considered indicative of complete gas film loss.

Stomatal aperture was evaluated under submergence conditions. For diurnal observations, fifteen plants were placed in an open field under full sunlight. Between 08:00 and 18:00, three mature leaves were collected every 30 min, and stomatal impressions were prepared using the nail polish imprint method (Weyers and Johansen, 1985). Stomatal aperture was examined under an upright fluorescence microscope (NIS-Elements AR). Stomata showing maximum opening were classified as “open, ”those at minimum opening as “closed, ”and intermediate states were also categorized as “open.” Stomatal observations were conducted at 10:00, corresponding to the diurnal peak of stomatal opening determined in preliminary trials, to minimize variability associated with circadian regulation.

During the submergence experiment, stomatal responses were monitored at 10:00 on days 1, 3, 5, and 7 post-submergences. For each treatment, one fully expanded mature leaf was randomly collected from three plants. After gently blotting the leaf surface with absorbent paper, stomatal impressions were immediately prepared using the nail polish imprint method. A thin layer of transparent nail polish was applied to the leaf surface and allowed to dry. The dried film was then carefully peeled off using transparent adhesive tape and mounted on a glass slide. Stomatal observations were conducted on the central lamina region, avoiding major veins, under an upright fluorescence microscope (NIS-Elements AR). At least five fields per leaf and three biological replicates were analyzed. This method follows standard protocols described in previous studies (Pathoumthong et al., 2023).

### 2.4 Plant growth assessment

Plants were assessed for morphological traits before, during, and after submergence. Prior to submergence, main stem length, total leaf number, and number of green leaves were recorded for each plant. During submergence, total leaf number was recorded daily at 10:00. After submergence, main stem length, number of newly emerged leaves, and number of green leaves were measured in the four treatment groups. In addition, plants from the baseline group were used to separately collect leaves, stems, and roots, which were oven-dried at 60 °C in a forced-air oven for subsequent non-structural carbohydrate analysis. At the end of the treatments, all plants from both the submerged and unsubmerged groups were separated into leaves, stems, and roots, and oven-dried at 60 °C in a forced-air oven.

### 2.5 Non-structural carbohydrate analysis

Non-structural carbohydrates (NSCs) were quantified in dried leaves, stems, and roots of plants. Fine powder samples were prepared with a ball mill (WS-MM200, Retsch, Haan, Germany). Soluble sugars and starch were extracted and determined following a modified anthrone–sulfuric acid method (Zhang, 2003; Lei et al., 2014).

Soluble sugars were extracted from 0.01 g of plant dry weight using 5 mL of 80% (v/v) ethanol. The extraction was performed at 80 °C and repeated twice. The supernatants were combined and used for soluble sugar determination. The ethanol-insoluble fraction of the same residue was subsequently extracted twice with ultrapure water at 80 °C for 40 min and diluted to 50 ml. Starch remaining in the residue was hydrolyzed with 5 mL 6 mol L^-^¹ HCl, and the hydrolysate was filtered and diluted to 100 ml.

For quantification, 1 ml of each diluted extract (ethanol-soluble sugars, ethanol-insoluble sugars, and starch hydrolysate) was mixed with 5 ml anthrone–sulfuric acid reagent (0.1 g anthrone in 100 ml of 75% (v/v) H_2_SO_4_) and heated at 100 °C for 10 min. Absorbance was measured at 625 nm using a spectrophotometer (UV-2700, Shimadzu, Japan) and concentrations were calculated from a glucose calibration curve. Starch content was determined by multiplying the concentration of hydrolytic soluble sugars by a conversion factor of 0.9. Total soluble sugars were calculated as the sum of ethanol-soluble and ethanol-insoluble sugars, and NSCs were defined as the sum of total soluble sugars and starch.

### 2.6 Target central carbon metabolomic analysis

To obtain the absolute concentrations of metabolites in specific metabolic pathways (glycolysis, the tricarboxylic acid cycle, and the pentose phosphate pathway) in leaves of *Cynodon dactylon* with and without gas films, a targeted metabolomics assay was performed. A total of 57 targeted metabolites were detected; the detailed names of these metabolites are provided in **Supplementary Methods S1**. The leaves were treated with 0.05% Triton, then submerged for 3 and 7 days, finally collected after liquid nitrogen treatment. Use the Waters ACQUITY I-Class ultra-high performance liquid chromatography instrument, the ACQUITY UPLC BEH Amide 1.7 μm (1.7 μm × 2.1*150mm, Waters) liquid chromatography column was used for the chromatographic separation of the target compounds (parameters of column oven temperature set at 40 °C, sample plate set at 10 °C, and injection volume of 1 μL). Molecular mass analysis was conducted using the SCIEX QTRAP 6500+ quadrupole mass spectrometer equipped with the IonDrive Turbo V ESI ion source (ion source parameters: Curtain Gas = 20 psi, IonSpray Voltage = +5500 V, -4500 V, Temperature = 500 °C, Ion Source Gas 1 = 50 psi, Ion Source Gas 2 = 55 psi). Mobile phase flow rate: 0.4 mL/min. Gradient elution program: 0–1 min, 10% B; 1–7 min, 10%–50% B; 7–11 min, 50%–90% B; 11–14 min, 90% B; 14–15 min, 90%–10% B. The brief experimental procedure for determining the levels of targeted metabolites is as follows: add 500 microliters of methanol solution to the dried sample tube and vortex for 1 minute to mix. Centrifuge at 12, 000 revolutions per minute for 10 minutes at 4 °C, collect all the supernatants and transfer them to a new 2-milliliter centrifuge tube for concentration and drying. Then, precisely add 150 microliters of 2-chloro-L-phenylalanine solution (4 ppm) to re-dissolve the sample. Filter the supernatant through a 0.22-micrometer filter membrane and transfer the filtrate to a detection bottle for LC-MS analysis. Each treatment has three replicates.

### 2.7 Statistical analysis

Stomatal opening percentage was calculated as:

$$\text{The Percentage of stomatal opening}=\frac{\text{Number of open stomata per unit area}}{\text{Total number of stomata per unit area}}\times 100\%$$

Growth rate of the main stem was expressed as:

$\text{RGR}\;(\text{cm}\cdot {\text{cm}}^{-1}\cdot {\text{d}}^{-1})=\frac{{\text{lnA}}_{2}–{\text{lnA}}_{1}}{{\text{t}}_{2}–{\text{t}}_{1}}$

Where A_1_ is the initial main stem length and A_2_ is the main stem length after treatment; t_1_ and t_2_ represent the start and end of the submergence period, respectively.

Statistical analyses were performed using IBM SPSS Statistics (IBM Corp., Armonk, NY, USA). One-way ANOVA with Duncan *post hoc* test was used to investigate the difference among treatments. Figures were generated using Origin 2018 and Microsoft Office 2019.

## 3 Results

### 3.1 Leaf gas films and stomatal behaviors of *Cynodon dactylon* under complete submergence

In this study, contact angle measurements exceeding 150°confirmed the super hydrophobicity of the leaf cuticle, suggesting that epicuticular waxes form a stable solid–gas–liquid interface that facilitates gas retention (**Table 1**; **Supplementary Figure 1**).

**Table 1:** Changes in contact angle between *C. dactylon* leaf surfaces and water droplets during submergence.

Duration of submergence (day)	Contact angle of the water droplet (°)	
	Adaxial	Abaxial
1	154.25 ± 6.22a	140.60 ± 5.06a
7	103.13 ± 5.95b	84.95 ± 2.42b

Values represent means ± SE (*n* = 5). Values sharing different letters within a column are significantly different (*P* < 0.05) according to Student’s t-test (independent samples).

**Figure 1:**
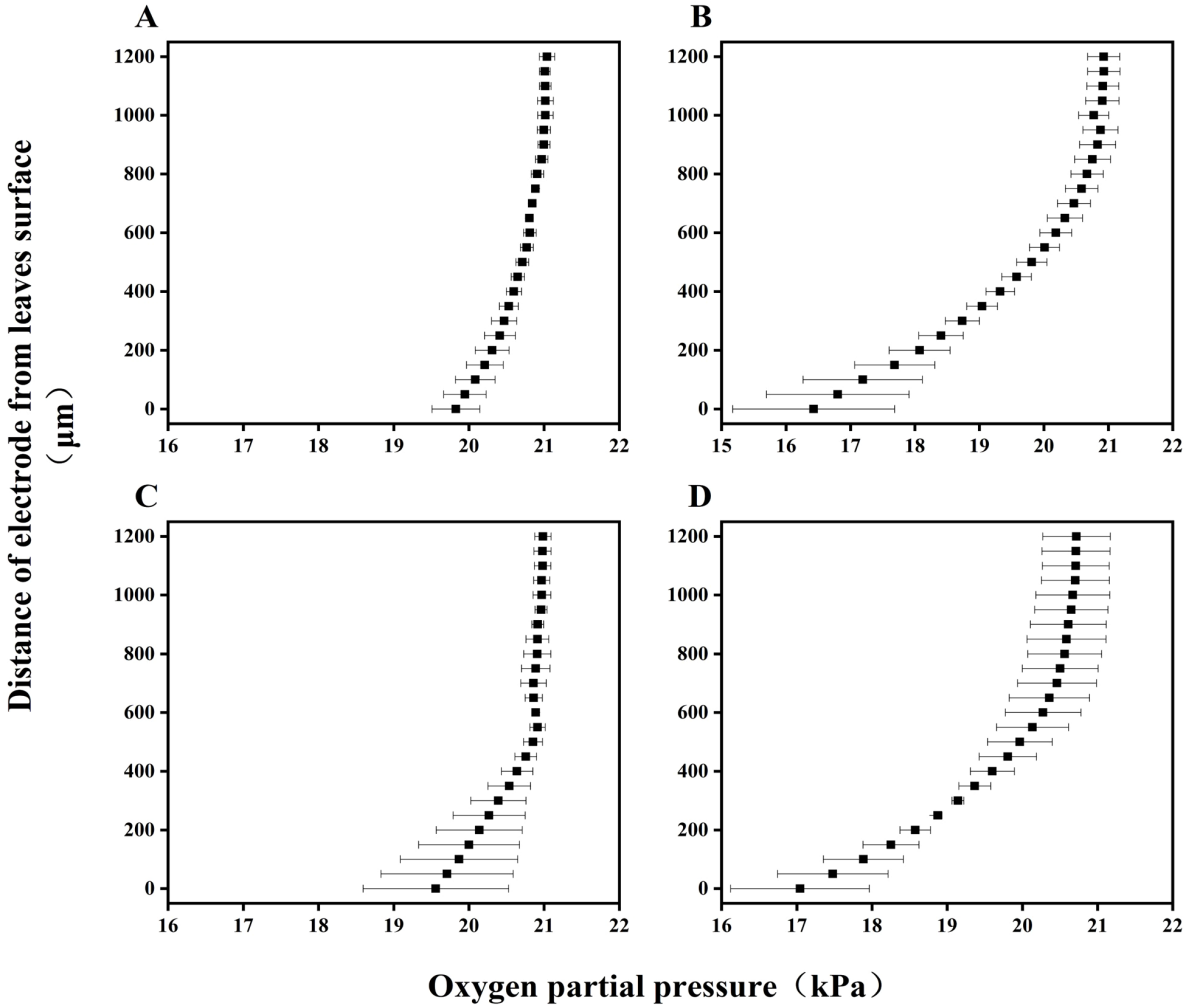
Profiles of oxygen partial pressure above the leaf surface of *C. dactylon* submerged under water after different treatments: **(A)** adaxial surface with water treatment; **(B)** adaxial surface with Triton X-100 treatment; **(C)** abaxial surface with water treatment; and **(D)** abaxial surface with Triton X-100 treatment. Values represent means ± SE (*n* = 3).

The functional significance of these gas films was evident from oxygen partial pressure profiles near submerged leaves. Leaves with intact surfaces (water treatment) maintained higher oxygen levels than those treated with Triton X-100, which removes epicuticular wax. On the adaxial surface, oxygen partial pressures were 20.2 kPa (treated by water) versus 17.7 kPa (treated by Triton X-100); on the abaxial surface, 19.8 kPa versus 17.9 kPa, respectively (**Figure 1**).

**Figure 2:**
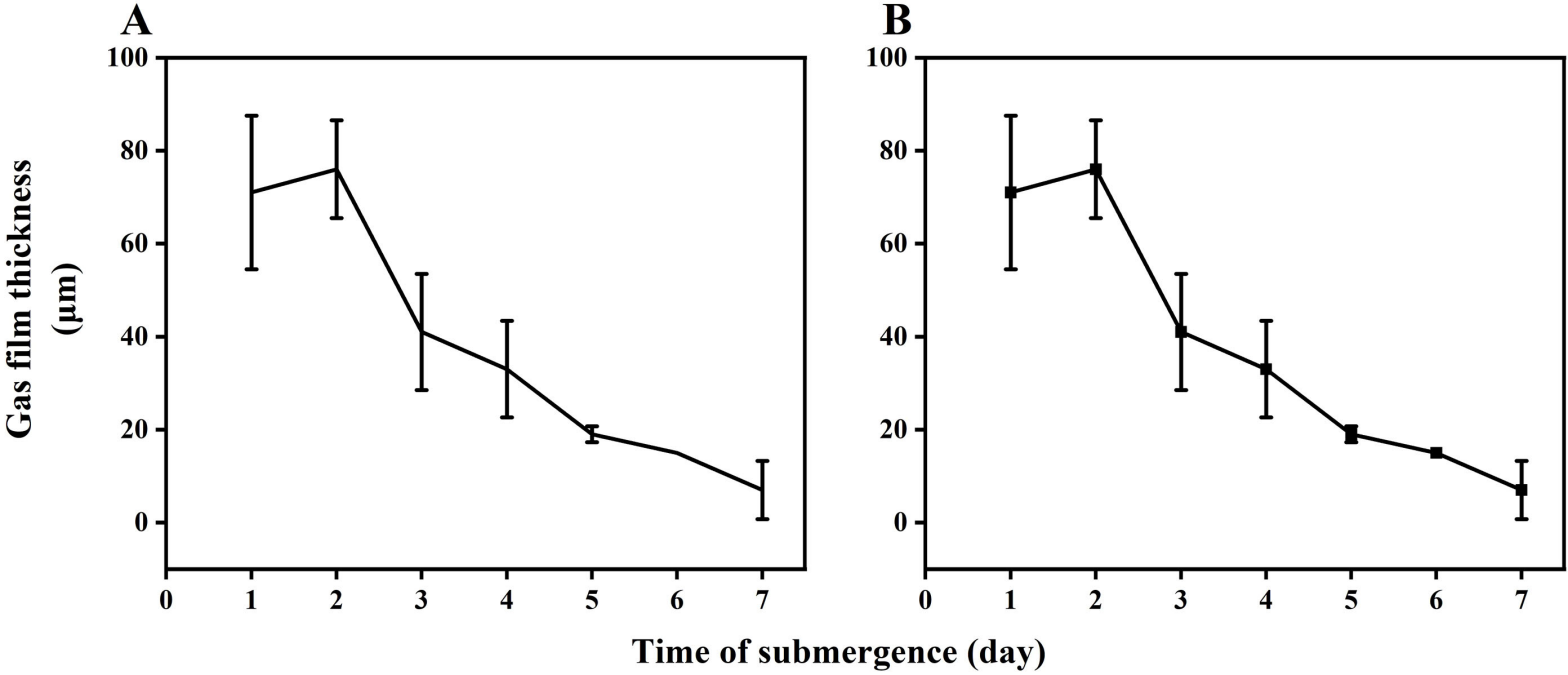
Changes in gas film thickness on the surfaces of *C. dactylon* leaves with increasing flooding duration: **(A)** adaxial surface and **(B)** abaxial surface. Values represent means ± SE (*n* = 3).

Moreover, it was found that gas film thickness declined progressively during submergence. On day 1, the thickness of films was ~70 µm (adaxial) and ~60 µm (abaxial), decreasing by 5–20 µm per day. By day 7, gas films had completely disappeared (**Figure 2**). The transient nature of these films suggests that *C. dactylon* relies on them primarily as a short-term acclimation mechanism during early flooding.

**Figure 3:**
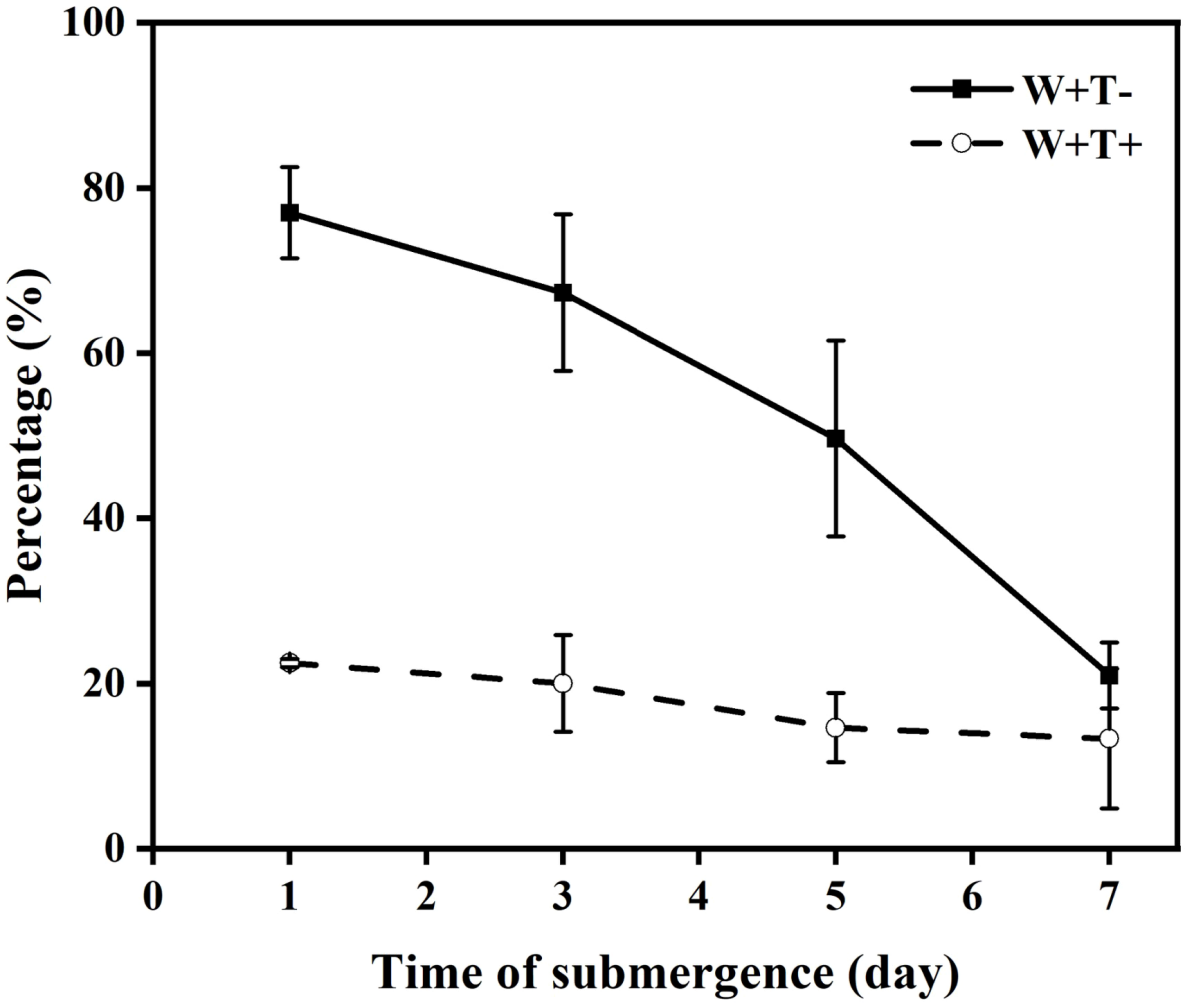
Changes in stomatal opening ratio of *C. dactylon* leaves during submergence. W+T− indicates complete submergence with gas film retained, and W+T+ indicates complete submergence with gas film removed. Values represent means ± SE (*n* = 3).

Stomatal behavior was assessed at the onset of treatments, and no significant differences in stomatal density were observed among treatments in *C. dactylon* (206.4 mm^-^² in W+T−, 196.7 mm^-^² in W+T+). During submergence, stomata in the W+T− group were initially widely open (~80% on day 1) but gradually closed, with the proportion of open stomata declining to ~20% by day 7. In contrast, stomata in the W+T+ group remained only partially open (~20%) throughout the submergence period (**Figure 3**).

### 3.2 Growth and biomass responses

**Figure 4:**
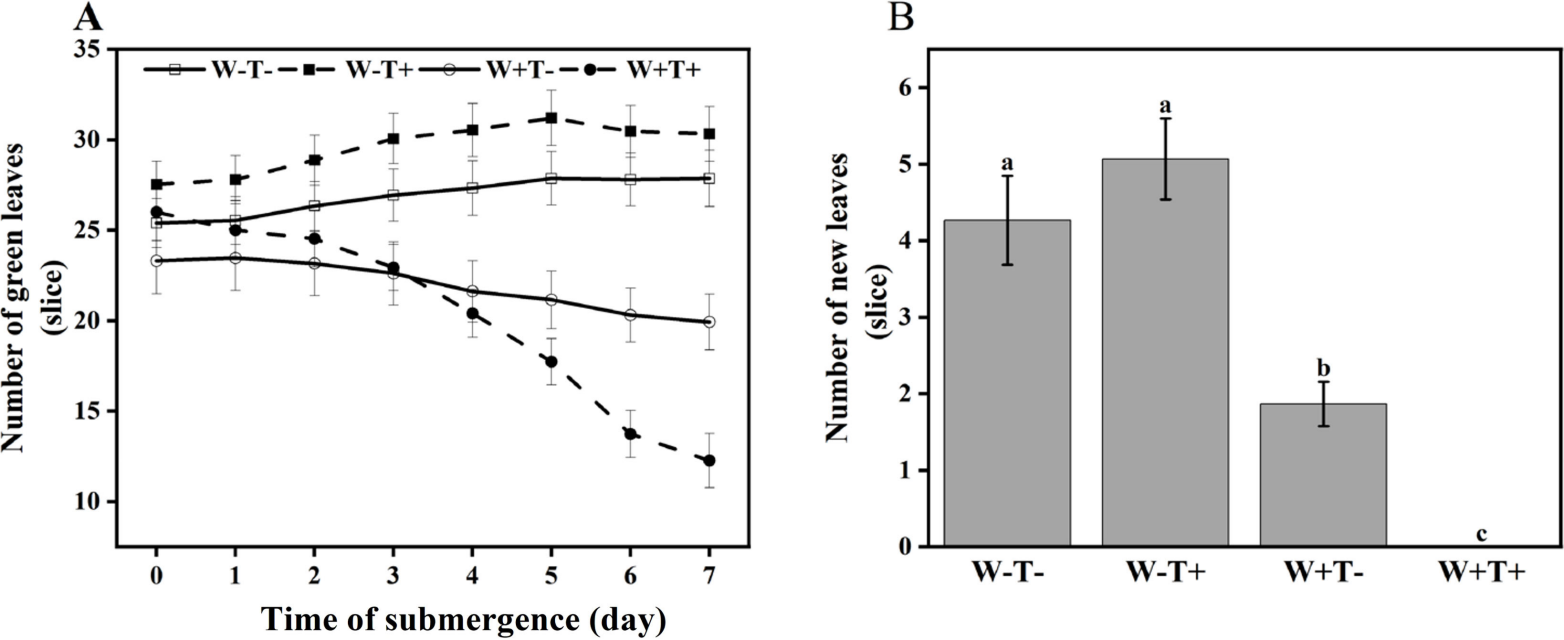
Changes in the number of **(A)** green leaves and **(B)** newly emerged leaves of *C. dactylon* during flooding. Values represent means ± SE (*n* = 15). Treatments are as follows: W−T−, unsubmerged with gas film retained; W−T+, unsubmerged with gas film removed; W+T−, completely submerged with gas film retained; and W+T+, completely submerged with gas film removed. One-way ANOVA was used to test differences in the number of newly emerged leaves among treatments. Different letters indicate significant differences (*p* < 0.05).

In this study, the changes of leaves were recorded during treatment. At the start of the experiment, green leaf number ranged from 23 to 27 across all treatments. In the unsubmerged groups (W−T− and W−T+), leaf numbers increased gradually over time, with no marked difference between the two treatments. In contrast, both submerged groups exhibited a decline in green leaf number, but with distinct patterns. In W+T− plants, leaf numbers decreased only slightly, remaining above 20 even after 7 d of submergence. By contrast, W+T+ plants showed a sharp reduction, beginning from day 3–4 and peaking at day 6, with only ~13 green leaves remaining by day 7 (**Figure 4A**). Moreover, it was found that there were newly produced leaves during the treatment period. The number of new leaves did not differ significantly between the unsubmerged groups (W−T− and W−T+), both producing 4–5 new leaves (*p* > 0.05). In submerged plants, however, new leaf production was strongly suppressed: W+T− plants developed two new leaves, whereas W+T+ plants produced none, a significant difference between the two groups (*p* < 0.05). Comparisons between gas film–retaining and submerged conditions also showed significant differences. W−T− plants produced more new leaves than W+T− plants (*p* < 0.05), and W−T+ plants produced more than W+T+ plants (*p* < 0.05). These results indicate that submergence strongly restricts new leaf formation, and that the presence of gas films partially influences this response (**Figure 4B**).

**Figure 5:**
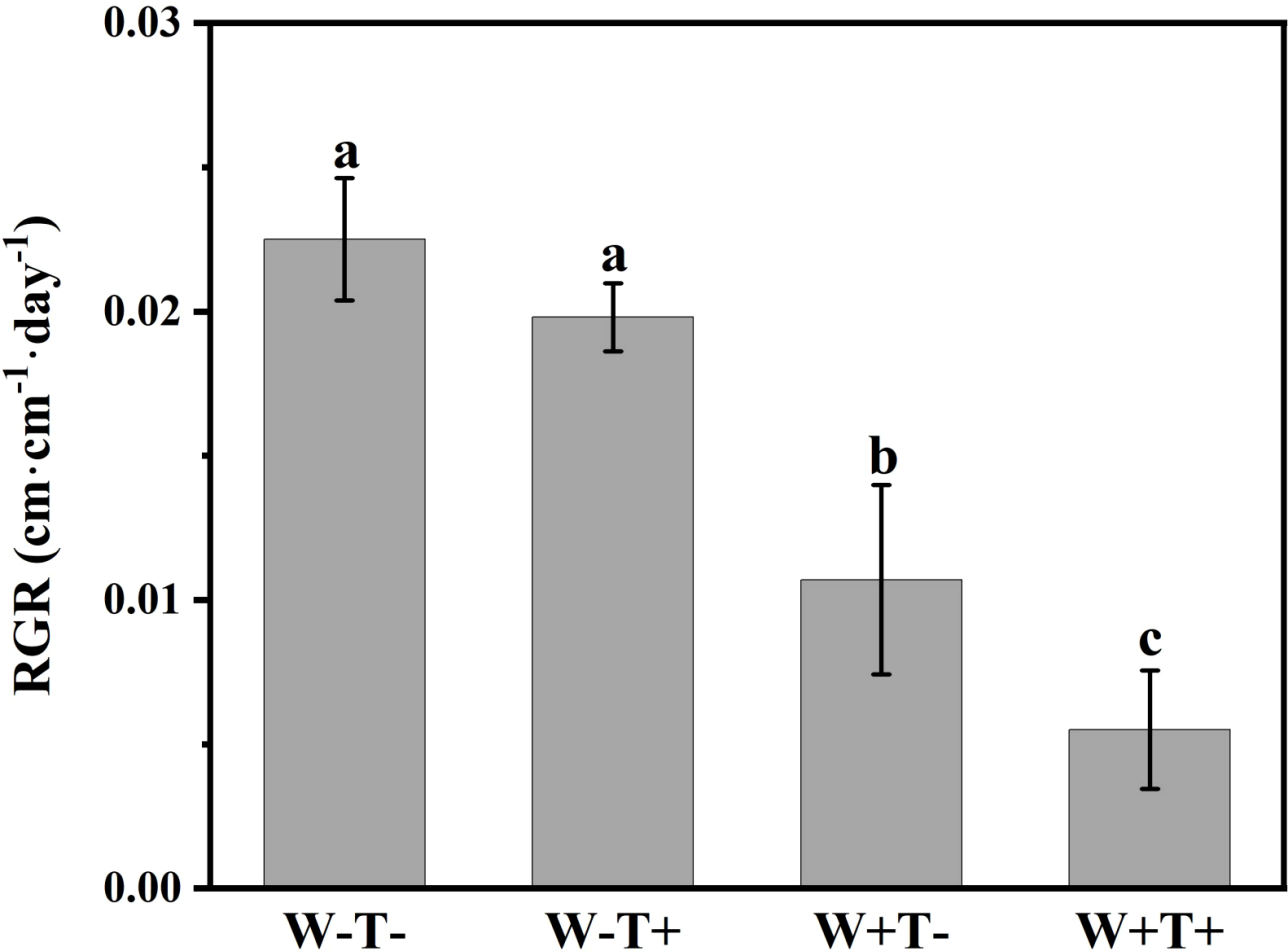
Relative growth rate (RGR; cm˙ cm^-^¹˙ d^-^¹) of the main stem of *C. dactylon* under four different treatments. Values represent means ± SE (*n* = 15). Treatments are as follows: W−T−, unsubmerged with gas film retained; W−T+, unsubmerged with gas film removed; W+T−, completely submerged with gas film retained; and W+T+, completely submerged with gas film removed. One-way ANOVA was used to test differences in RGR among treatments. Different letters indicate significant differences (*p* < 0.05).

During submergence, stem growth of *C. dactylon* also showed clear treatment effects. Relative stem growth rates did not differ significantly between the unsubmerged groups (W−T− and W−T+) (*p* > 0.05). Among submerged plants, W+T− exhibited a rate of ~0.01 cm˙cm^-^¹˙d^-^¹, which was significantly higher than the ~0.007 cm˙cm^-^¹˙d^-^¹ observed in W+T+ plants (*p* < 0.05). Comparisons across submergence treatments further showed that W−T− plants had significantly higher growth rates than W+T− plants (*p* < 0.05), and W−T+ plants also exceeded W+T+ plants (*p* < 0.05) (**Figure 5**).

### 3.3 Non-structural carbohydrate responses

**Figure 6:**
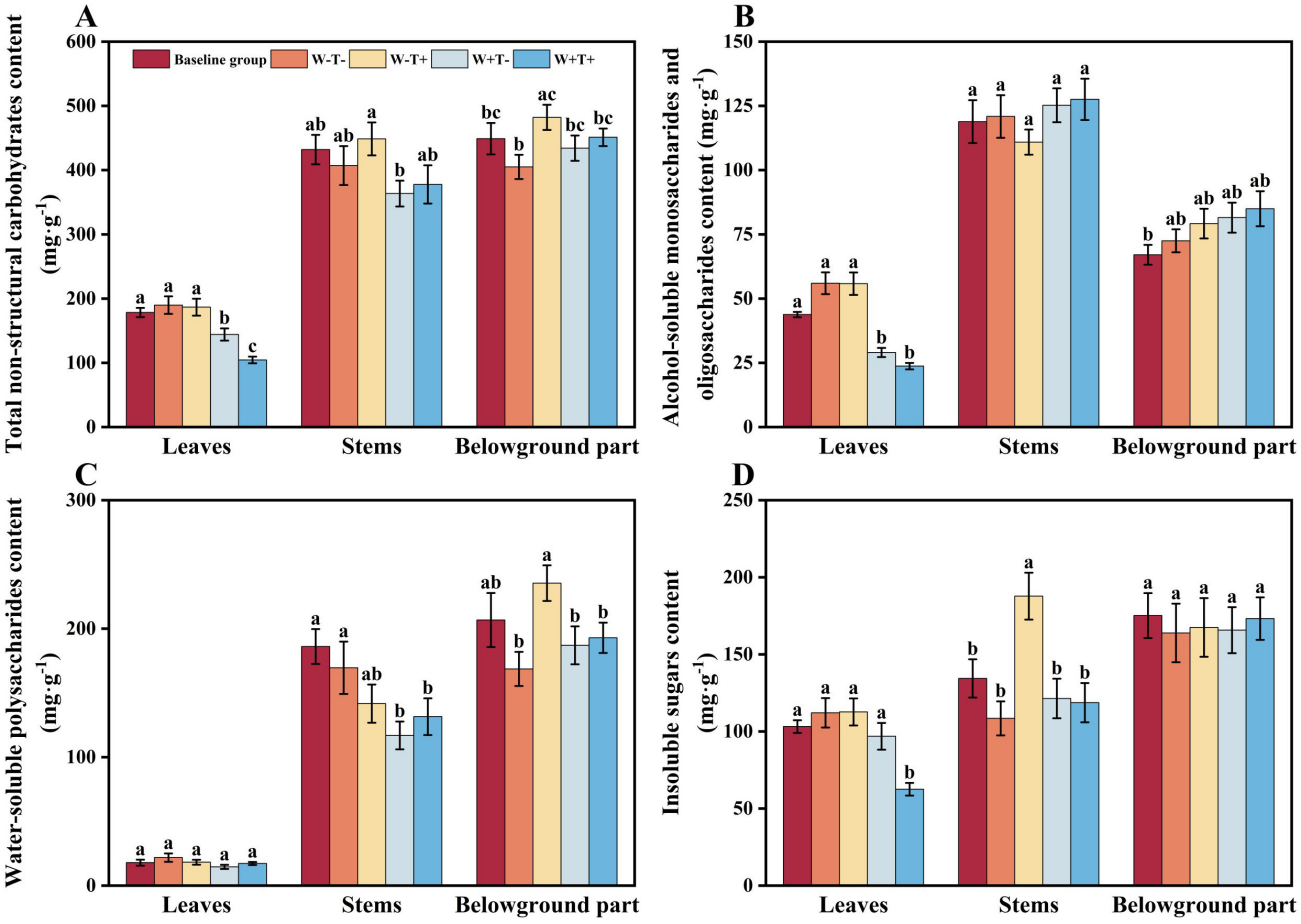
Changes in the contents of **(A)** total non-structural carbohydrates, **(B)** alcohol-soluble monosaccharides and oligosaccharides, **(C)** water-soluble polysaccharides, and **(D)** insoluble sugars in the leaves, stems, and belowground parts (root + rhizomes) of *Cynodon dactylon* under different treatments. Values represent means ± SE (*n* = 15). Baseline group indicates the control with no treatment applied. Treatments are as follows: W−T−, unsubmerged with gas film retained; W−T+, unsubmerged with gas film removed; W+T−, completely submerged with gas film retained; and W+T+, completely submerged with gas film removed. One-way ANOVA was used to test differences in carbohydrate contents among treatments. Different letters indicate significant differences (*p* < 0.05) in a particular type of carbohydrate within the same organ under different treatments.

NSC concentrations differed among organs and treatments in *C. dactylon*. In leaves, both unsubmerged groups (W−T− and W−T+) maintained significantly higher NSC concentrations than submerged groups (W+T− and W+T+) (*p* < 0.05), with no difference between the two unsubmerged groups (*p* > 0.05). Among submerged plants, however, W+T− retained significantly more NSC than W+T+ (*p* < 0.05) (**Figure 6A**). In stems, submergence tended to reduce NSC concentrations relative to unsubmerged controls, although the decrease was not statistically significant (*p* > 0.05). No significant differences were detected either between the two unsubmerged groups or between the two submerged groups (*p* > 0.05) (**Figure 6A**). In roots, NSC concentration showed no significant differences between W+T− and W+T+ (*p* > 0.05) (**Figure 6A**).

Overall, submergence markedly constrained leaf NSC reserves, while gas film retention alleviated this decline under water. In contrast, stem and root NSC concentrations were less responsive to treatments, suggesting organ-specific sensitivity to submergence stress and existence of leaf gas film.

Non-structural carbohydrates in *C. dactylon* consisted of soluble sugars (alcohol-soluble monosaccharides and oligosaccharides, and water-soluble polysaccharides) and insoluble sugars, whose concentrations varied among organs and treatments. In leaves, alcohol-soluble monosaccharides and oligosaccharides did not differ significantly among the baseline group and the two unsubmerged treatments (W−T− and W−T+), nor between the two submerged treatments (W+T− and W+T+) (*p* > 0.05). However, both submerged groups exhibited significantly lower concentrations than the unsubmerged groups (*p* < 0.05) (**Figure 6B**). Water-soluble polysaccharides showed no significant differences across treatments (*p* > 0.05) (**Figure 6C**). Leaf insoluble sugar concentration was largely unchanged among treatments, except that the W+T+ group exhibited a significantly different value compared with other groups (*p* < 0.05) (**Figure 6D**).

In stems, alcohol-soluble monosaccharides and oligosaccharides were unaffected by treatments (*p* > 0.05) (**Figure 6B**). Water-soluble polysaccharides did not differ among the baseline group and the two unsubmerged treatments, nor between the two submerged treatments (*p* > 0.05) (**Figure 6C**). Insoluble sugar concentrations showed no difference between the baseline group and W−T− (*p* > 0.05), but were significantly different from W−T+ (*p* < 0.05). No significant differences were detected between the two submerged groups (*p* > 0.05) (**Figure 6D**).

In roots, alcohol-soluble monosaccharides and oligosaccharides were comparable across treatments, except for the baseline group (*p* < 0.05) (**Figure 6B**). Water-soluble polysaccharides remained stable, apart from a significant difference in the W−T+ group (*p* < 0.05) (**Figure 6C**). Insoluble sugars did not differ significantly across treatments (*p* > 0.05) (**Figure 6D**).

Collectively, these results indicated that submergence reduced the accumulation of soluble sugars in leaves, whereas stems and roots maintained relatively stable carbohydrate pools.

### 3.4 Variations of central carbon metabolism

**Figure 7:**
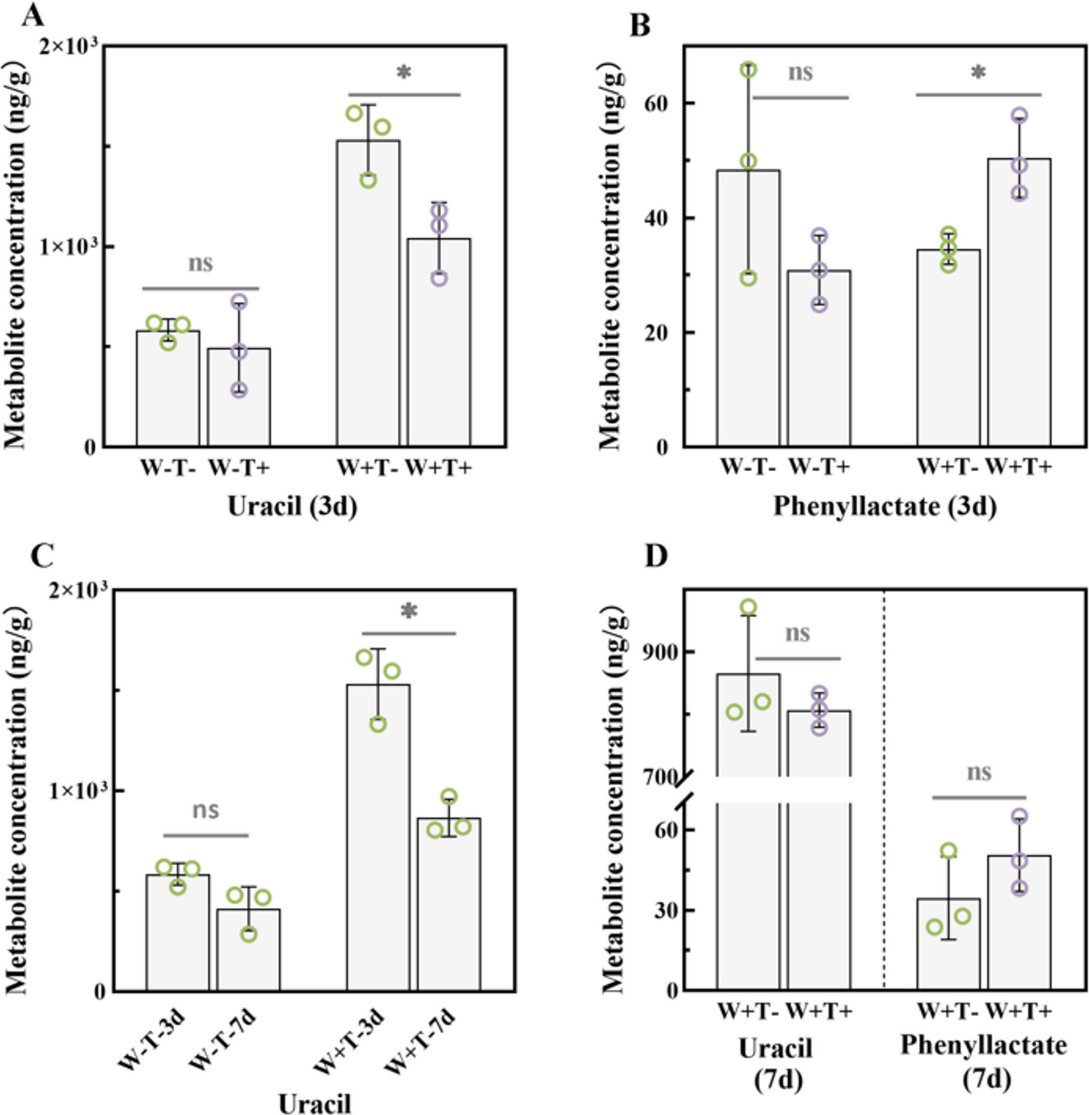
Absolute contents of uracil and phenyllactate in *C. dactylon* leaves. **(A)** Uracil content after 3 days of treatment; **(B)** phenyllactate content after 3 days of treatment; **(C)** uracil content after 3 and 7 days of treatment; and **(D)** uracil and phenyllactate contents after 7 days of treatment. W− indicates no flooding, W+ indicates submergence treatment, T− indicates no Triton X-100 treatment, and T+ indicates Triton X-100 treatment. Values represent means ± SD (*n* = 3). Treatments are as follows: W−T−, unsubmerged with gas film retained; W−T+, unsubmerged with gas film removed; W+T−, completely submerged with gas film retained; and W+T+, completely submerged with gas film removed. Independent-samples t-test was used to test differences in uracil and phenyllactate contents among treatments. “ns” indicates no significant difference, and asterisks denote significant differences (*p < 0.05) between groups.

**Figure 8:**
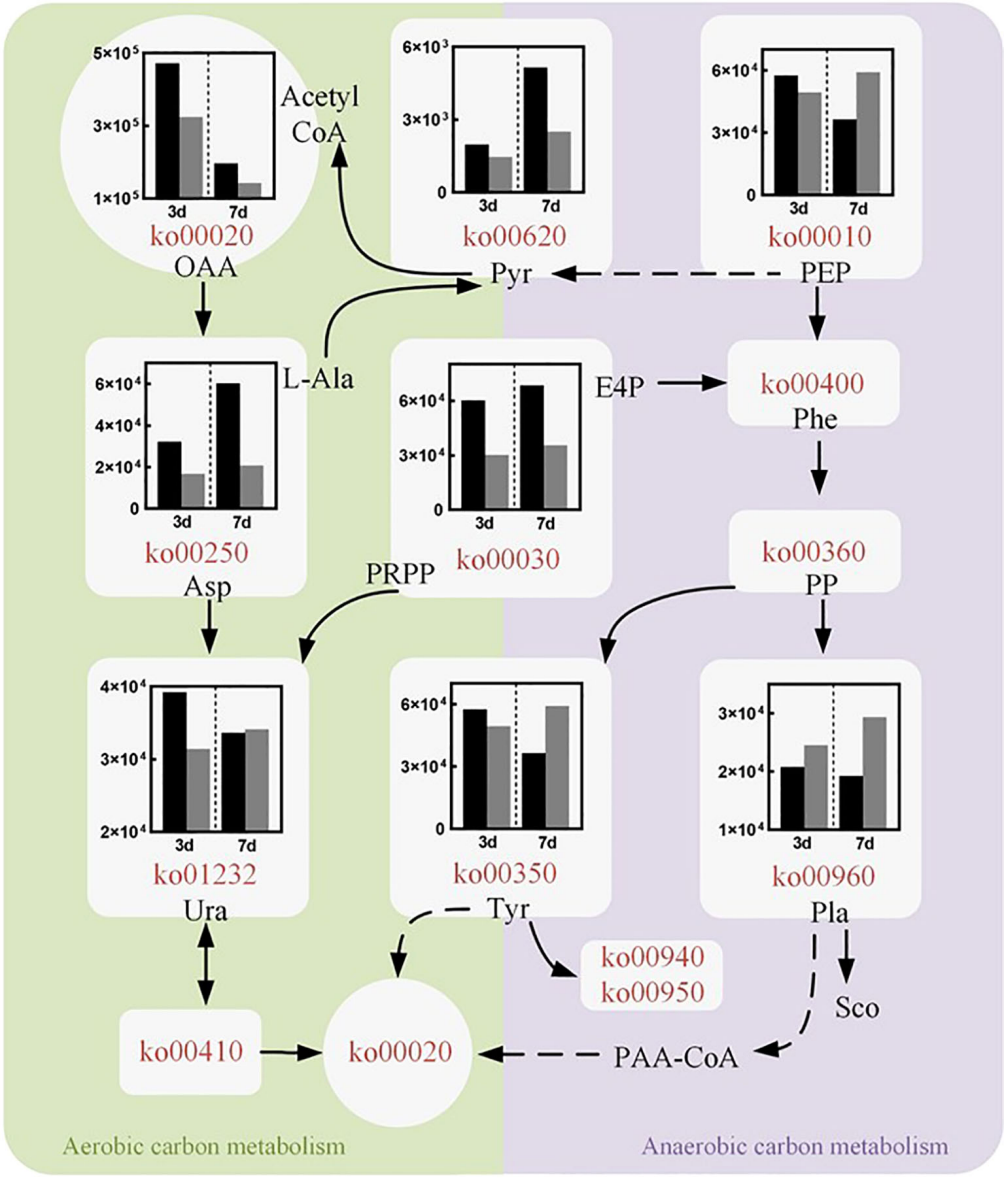
Proposed model of carbon metabolism in *C. dactylon* leaves influenced by gas films during submergence. KEGG pathway descriptions and column chart compositions are provided in **Supplementary Table 1**. Black columns represent leaves with gas films, and gray columns represent leaves without gas films. The black dashed line indicates the restricted flux of anaerobic carbon metabolism products entering the aerobic pathway. X-axis: treatment time; Y-axis: metabolite abundance in ng/g. The raw data of all metabolites are presented in **Supplementary Table 2**. OAA, oxaloacetate; Pyr, pyruvic acid; PEP, phosphoenolpyruvic acid; L-Ala, L-alanine; Asp, aspartic acid; PRPP, phosphoribosyl pyrophosphate; E4P, erythrose 4-phosphate; Phe, phenylalanine; PP, phenylpyruvate; Ura, uracil; Tyr, L-tyrosine; Pla, phenyllactate; PAA-CoA, phenyllactyl-CoA; Sco, scopolamine.

PCA and OPLS-DA analyses revealed clear metabolic divergence between submerged plants with and without gas films (**Supplementary Figures 2**, **3**). After 3 days of submergence, W+T− plants exhibited higher uracil but lower phenyllactate compared with W+T+ (**Figures 7A, B**; **Supplementary Figure 4**). In W+T−, uracil concentrations declined markedly between day 3 and day 7 (**Figure 7C**), indicating a progressive reduction in aerobic metabolism. Pathway mapping further demonstrated that W+T− plants on day 3 displayed enhanced flux through the TCA cycle, pentose phosphate pathway, and nucleotide biosynthesis (**Figure 8**), whereas W+T+ plants at day 7 showed an increased investment in secondary metabolism.

Together, these results suggest that gas films facilitate aerobic respiration and carbon metabolism during the early stages of submergence, thereby delaying the shift towards fermentative and secondary pathways. However, as gas films gradually deteriorate, their metabolic benefits diminish, forcing submerged plants to increasingly rely on less efficient anaerobic processes. This dynamic highlight gas film formation as a transient but ecologically significant trait that extends the window of aerobic energy production under complete submergence.

## 4 Discussion

Previous studies have demonstrated that gas films formed on the leaf surfaces of some flood-tolerant plants can facilitate underwater gas exchange and enhance photosynthetic activity (Colmer and Pedersen, 2008b; Winkel et al., 2011; 2013; 2017; Verboven et al., 2014; Mori et al., 2019). However, the functional significance of gas films in regulating carbon metabolism under submergence remains poorly understood. In this study, we show that leaves of *C. dactylon* develop distinct gas films when submerged. These films sustain aerobic metabolism, improve carbohydrate utilization efficiency, and mitigate damage caused by flooding stress.

### 4.1 Gas films may contribute to improved physiological performance under submergence

During submergence, leaves of *C. dactylon* developed distinct gas films, which may contributed to improved physiological performance. Based on measurements of contact angles between leaf surfaces and water droplets, together with oxygen partial pressure profiles in the surrounding water, it was evident that gas films formed on leaves under complete submergence (**Supplementary Figure 1**; **Figure 1**). Similar observations have been reported for several riparian grasses that produce gas films upon flooding (Colmer and Pedersen, 2008a; Verboven et al., 2014; Winkel et al., 2016). These films arise from the combined effects of a hydrophobic cuticular wax layer and specific epidermal microstructures, enabling a thin gaseous layer to persist on the leaf surface when in contact with water (Riederer and Schreiber, 2001; Colmer and Pedersen, 2008a). Additional leaf surface traits that may influence gas film remain to be elucidated. The presence of gas films not only established a physical interface between leaves and the surrounding water but also significantly increased the proportion of open stomata (**Figure 3**). Previous studies have shown that gas films retain near-surface air pathways, thereby maintaining stomatal gas exchange and enhancing oxygen diffusion into leaf tissues (Colmer and Pedersen, 2008b; Winkel et al., 2014). Consistent with these findings, we observed higher oxygen content in leaves with gas films compared to those without. This mechanism is particularly critical under hypoxic stress, as oxygen from the water can diffuse through the gas film into leaf tissues and be transported to cells to support aerobic respiration. Such functions align with our observations of elevated leaf surface oxygen partial pressures, greater stomatal opening, and higher early-stage carbon aerobic metabolic activity in gas film–bearing leaves.

Carbohydrate status is closely associated with stress tolerance, with higher carbohydrate reserves often conferring greater resilience (Koch, 1996; Figueroa and Lunn, 2016; Pramanik et al., 2016). Our results showed that leaves with gas films maintained higher levels of non-structural carbohydrates (NSCs) and insoluble sugars under submergence (**Figure 6**). This suggests that gas film formation enhances flooding tolerance by sustaining carbon reserves. This mechanism was further supported by the higher number of retained and newly developed leaves, as well as faster main stem elongation, observed in plants that maintained gas films (**Figures 4**; **5**). Together, these results indicate that gas film formation substantially improves flooding tolerance in *C. dactylon*.

As the primary photosynthetic organs, leaves play a crucial role in the post-flooding recovery of submerged plants (Mommer and Visser, 2005; Yeung et al., 2018). Although photosynthetic performance was not directly measured, the retention of a greater leaf area under submergence may facilitate rapid recovery of photosynthesis following de-submergence. The retention of leaf area under submergence may provide a structural basis for post-submergence recovery; however, this was not directly assessed in the present study. Compared with species whose leaves are more prone to submergence damage, *C. dactylon* may displayed rapid regrowth after de-submergence, because allowing it to occupy broader ecological niches and establish dominance in riparian habitats.

### 4.2 Gas films support central carbon metabolism through oxygen supply

Central carbon metabolism, including glycolysis, the tricarboxylic acid (TCA) cycle, and the pentose phosphate pathway (PPP), underpins energy supply and plant growth (Wu et al., 2023). Our metabolomic analyses revealed that by day 3 of submergence, glycolytic activity was comparable between plants with and without leaf gas films, but striking differences emerged in the PPP. Under submergence, plants retaining gas films-maintained PPP activity, leading to sustained production of phosphoribosyl pyrophosphate (PRPP) and thereby supporting uracil biosynthesis. Within the pyrimidine metabolic network, uracil serves not only as a precursor for UTP and CTP but also as a central node linking nucleotide turnover, amino acid metabolism, and energy metabolism (Sato and Ashihara, 2008; Skinner et al., 2023). Uracil is the first stable pyrimidine base synthesized, and its derivatives (UMP → UDP → UTP) form precursors for other pyrimidines such as CTP, placing uracil at a metabolic hub (Jones, 1980; Traut, 1994; Zrenner et al., 2006). UDP-sugars, such as UDP-glucose and UDP-galactose, are essential activated sugar donors in starch synthesis, cell wall formation, and polysaccharide modification (Kleczkowski et al., 2004; Bar-Peled and O’Neill, 2011);. Uracil is therefore considered a highly connected “hub metabolite” with broad upstream and downstream branches (Jeong et al., 2000; Boldt and Zrenner, 2003). Compared with adenine, guanine, or cytosine, uracil frequently emerges as the earliest and most stable pyrimidine base, underscoring its central role (Jones, 1980; Zrenner et al., 2006);.

Pyrimidine biosynthesis is dependent on mitochondrial function and oxygen as the terminal electron acceptor (Stasolla et al., 2003). Under hypoxia, the mitochondrial electron transport chain is impaired, dihydroorotate dehydrogenase (DHODH) becomes inactivated, and orotate cannot be oxidized, thereby restricting or blocking uracil synthesis (Löffler et al., 2005). Consequently, uracil biosynthesis is highly sensitive to cellular oxygen availability. Under low-oxygen conditions, restricted uracil synthesis leads to deficiencies in UMP/UTP, which in turn limit RNA/DNA synthesis and cell proliferation (Bailey-Serres et al., 2012). By contrast, under normoxia, efficient mitochondrial electron transport sustains DHODH activity and stable pyrimidine biosynthesis, supporting rapid growth and cell division (Vedel et al., 1999). In our study, the accumulation of uracil in submerged plants with gas films was associated with higher oxygen availability enabled sustained aerobic metabolism, which was consistent with their enhanced stem elongation and new leaf emergence compared with plants lacking gas films.

In contrast, plants without gas films accumulated phenyllactic acid, a derivative of phenylalanine that reflects a fermentative branch of amino acid metabolism (Huang et al., 2021; Ponzio et al., 2024). Under hypoxia, when the mitochondrial respiratory chain is blocked, NADH cannot be efficiently reoxidized via oxidative phosphorylation (Plaxton and Podestá, 2006). To sustain glycolysis, cells must regenerate NAD^+^ through alternative electron-accepting reactions. The reduction of phenylpyruvate to phenyllactic acid consumes NADH and regenerates NAD^+^, thereby maintaining glycolytic flux (Evans and Guy, 2004). Thus, the accumulation of phenyllactic acid represents an auxiliary strategy for energy maintenance under oxygen limitation. In addition, phenyllactic acid exhibits antimicrobial properties, including antifungal activity (Chaudhari and Gokhale, 2016), and may provide ecological advantages by enhancing pathogen defense (Xu et al., 2024). More broadly, the accumulation of secondary metabolites during stress has been widely documented as part of plant defense strategies (Dong and Lin, 2021; Singh et al., 2021; Yan et al., 2021). As the most representative metabolite of anaerobic respiration, lactate showed little difference between the two groups at 3 days of submergence. However, when submergence was prolonged to 7 days, the lactate content in leaves without gas films was about 30% higher than that in leaves with gas films, indicating more severe hypoxia in plants lacking gas films (**Supplementary Figure 5**). In this study, the increase in phenyllactic acid and lactate in leaves of plants without gas films under submergence reflected a metabolic shift toward anaerobic energy metabolism and secondary metabolic pathways (**Figures 7**, **8**).

We also observed that gas films on *C. dactylon* leaves were transient, disappearing by day 7 of submergence (**Figure 2**). This decline coincided with a reduction in TCA cycle activity, highlighting the critical role of gas films in sustaining aerobic metabolism during the early stages of submergence. Plants that retained gas films early in submergence maintained greater numbers of green leaves, exhibited superior stem growth, and preserved more favorable metabolic traits compared with those without gas films. These findings suggest that early aerobic support provides a metabolic advantage that facilitates rapid recovery once flooding recedes.

In summary, gas film formation in *C. dactylon* leaves regulates carbon metabolic pathways in a dynamic manner. During the early stages of submergence, gas films facilitate oxygen entry, sustain the TCA cycle, and promote energy production. Once gas films dissipate, metabolism shifts toward anaerobic and secondary pathways, maintaining cellular survival and defense. This flexible redistribution of carbon metabolism reflects an adaptive strategy that underpins the flooding tolerance of *C. dactylon*.

### 4.3 Duration of gas films on *C. dactylon* leaves and their ecological significance

In this study, gas films formed on *C. dactylon* leaves under submergence were maintained only for a limited period (approximately 7 days). Previous reports have also shown that gas films in many submerged plants are short-lived, typically persisting for 0–11 days (Winkel et al., 2016). Although transient, these structures confer significant ecological advantages. Natural flooding events in riparian zones are often brief, with submergence durations rarely exceeding one week (Colmer and Voesenek, 2009; Bailey-Serres et al., 2012). Within this critical but limited window, gas films provide an immediate aerobic interface that supports mitochondrial respiration and the tricarboxylic acid (TCA) cycle, thereby alleviating the energy crisis caused by oxygen deprivation. Moreover, gas films help maintain green, photosynthetically active tissues during the initial flooding phase, ensuring that plants retain the capacity for carbon assimilation and providing essential resources for post-flood recovery and regeneration (Bailey-Serres et al., 2012; Voesenek and Bailey-Serres, 2015);. This function is particularly important in turbid floodwaters, where light penetration is severely reduced (Grubbs and Taylor, 2004; Keddy, 2010). Thus, any mechanism that enhances oxygen acquisition and efficient carbohydrate utilization during submergence is crucial. By providing early-phase relief, gas films may improve the competitive advantage of *C. dactylon* in habitats with fluctuating water levels, such as reservoir drawdown zones, ultimately supporting individual survival and population persistence.

Despite their ecological importance, little is known about how leaf surface gas films are maintained in submerged environments or why they eventually dissipate in some species. In riparian habitats, plants typically experience high light and strong air circulation before flooding, leading to dry leaf surfaces with minimal microbial colonization (Arya et al., 2021). Upon submergence, however, leaf surfaces are exposed to saturated humidity; even within gas films, relative humidity approaches 100%. Such conditions favor microbial attachment and growth, which can lead to the formation of biofilms (Lindow and Brandl, 2003; Vorholt, 2012). Because biofilms are hydrophilic, their establishment may disrupt leaf surface hydrophobicity and cause gas films to collapse. Based on this, we hypothesize that microbial biofilms may influence the persistence of leaf gas films by altering surface wettability; however, this remains speculative and was not directly tested in this study. Future studies should therefore investigate the relationship between gas film longevity and microbial biofilm dynamics on submerged leaves.

Additionally, this study has several limitations. First, metabolic inferences are based on targeted metabolite profiling and do not include direct measurements of key physiological indicators such as ATP/ADP ratios, reactive oxygen species (ROS), or gene expression. Second, photosynthetic performance and post-submergence recovery were not directly assessed. Third, the study focused on a single species, which limits generalization across riparian plant communities. Future studies integrating physiological, transcriptomic, and ecological approaches will be necessary to further validate the proposed mechanisms.

## 5 Conclusions

**Figure 9:**
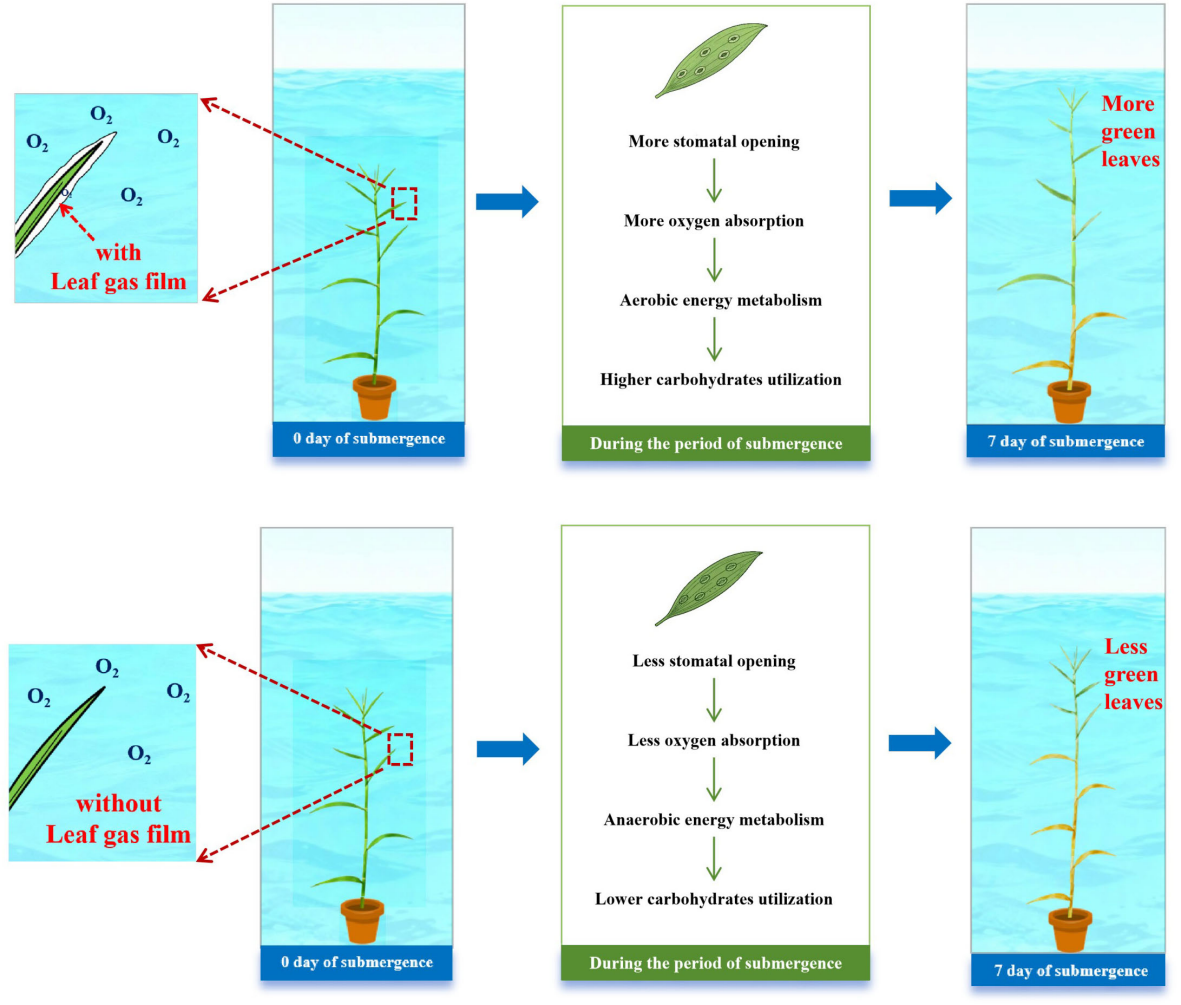
Conceptual model illustrating the potential role of leaf gas films in regulating physiological and metabolic responses of *Cynodon dactylon* under complete submergence. Plants retaining leaf gas films maintain greater stomatal opening and higher oxygen availability at the leaf surface, which may facilitate aerobic energy metabolism and more efficient carbohydrate utilization during submergence. In contrast, removal of gas films is associated with reduced stomatal opening, limited oxygen availability, and a shift towards anaerobic metabolism. These differences are reflected in leaf performance, with gas film retention associated with a higher number of green leaves, whereas plants lacking gas films show reduced leaf retention.

This study systematically elucidates the physiological and metabolic mechanisms underlying gas film formation on *C. dactylon* leaves during submergence (**Figure 9**). Gas films facilitated stomatal opening, improved oxygen availability, and supported aerobic respiration and central carbon metabolism, thereby alleviating energy imbalance under flooding stress. Metabolomic analyses further revealed that the presence of gas films sustained the flux of carbohydrates into the TCA cycle and pyrimidine (uridine) biosynthesis, maintaining energy supply. Once gas films dissipated, however, metabolism gradually shifted toward anaerobic and secondary pathways as plants adapted to oxygen limitation. Although gas films persisted only for a limited duration, their early protective role in energy metabolism and physiological performance provided critical support for flood tolerance in *C. dactylon*. Taken together, these findings provide mechanistic insight into how leaf-level traits may influence plant responses to flooding, while highlighting the need for integrative studies linking physiology, metabolism, and ecological outcomes.

## Data Availability

The raw data supporting the conclusions of this article will be made available by the authors, without undue reservation.

## Supplementary material

Supplementary Table 1 KEGG pathway descriptions and column chart compositions.

Supplementary Table 2 The quantified values of various central carbon metabolism metabolites.

Supplementary Figure 1 Water droplets on the leaf surfaces of *C. dactylon*.

Supplementary Figure 2 Principal component analysis (PCA) of samples.

Supplementary Figure 3 OPLS-DA analysis of samples.

Supplementary Figure 4 Volcano plot analysis of samples.

Supplementary Figure 5 Lactate content at 3 and 7 days of submergence.

## References

[B1] Aguiar F. C. Segurado P. Martins M. J. Bejarano M. D. Nilsson C. Portela M. M. . (2018). The abundance and distribution of guilds of riparian woody plants change in response to land use and flow regulation. J. Appl. Ecol. 55, 2227–2240. doi: 10.1111/1365-2664.13110 40046247

[B2] Allan C. J. Vidon P. Lowrance R. (2008). Frontiers in riparian zone research in the 21st century. Hydrol. Process. 22, 3221–3222. doi: 10.1002/hyp.7086 41531421

[B3] Andersen T. Pedersen O. (2002). Interactions between light and CO_2_ enhance the growth of Riccia fluitans. Hydrobiologia 477, 163–170. doi: 10.1023/A:1021007124604 41886696

[B4] Armstrong W. (1979). Aeration in higher plants. Adv. Bot. Res. 7, 225–332. doi: 10.1016/S0065-2296(08)60089-0

[B5] Arya G. C. Sarkar S. Manasherova E. Aharoni A. Cohen H. (2021). The plant cuticle: an ancient guardian barrier set against long-standing rivals. Front. Plant Sci. 12, 663165. doi: 10.3389/fpls.2021.663165 34249035 PMC8267416

[B6] Ayi Q. Zeng B. Liu J. Li S. van Bodegom P. M. Cornelissen J. H. C. (2016). Oxygen absorption by adventitious roots promotes the survival of completely submerged terrestrial plants. Ann. Bot. 118, 675–683. doi: 10.1093/aob/mcw051 27063366 PMC5055620

[B7] Bailey-Serres J. Lee S. C. Brinton E. (2012). Waterproofing crops: effective flooding survival strategies. Plant Physiol. 160, 1698–1709. doi: 10.1104/pp.112.208173 23093359 PMC3510103

[B8] Bar-Peled M. O’Neill M. A. (2011). Plant nucleotide sugar formation, interconversion, and salvage by sugar recycling. Annu. Rev. Plant Biol. 62, 127–155. doi: 10.1146/annurev-arplant-042110-103918 21370975

[B9] Boldt R. Zrenner R. (2003). Purine and pyrimidine biosynthesis in higher plants. Physiol. Plant 117, 297–304. doi: 10.1034/j.1399-3054.2003.00044.x 12654029

[B10] Bren L. J. (1993). Riparian zone, stream, and floodplain issues: a review. J. Hydrol. 150, 277–299. doi: 10.1016/0022-1694(93)90113-N

[B11] Chaudhari S. S. Gokhale D. V. (2016). Phenyllactic acid: a potential antimicrobial compound in lactic acid bacteria. J. Bacteriol. Mycol. Open Access 2, 121–125. doi: 10.15406/jbmoa.2016.02.00037

[B12] Colmer T. D. Pedersen O. (2008a). Underwater photosynthesis and respiration in leaves of submerged wetland plants: gas films improve CO_2_ and O_2_ exchange. New Phytol. 177, 918–926. doi: 10.1111/j.1469-8137.2007.02318.x 18086222

[B13] Colmer T. D. Pedersen O. (2008b). Oxygen dynamics in submerged rice (Oryza sativa). New Phytol. 178, 326–334. doi: 10.1111/j.1469-8137.2007.02364.x 18248586

[B14] Colmer T. D. Voesenek L. A. C. J. (2009). Flooding tolerance: suites of plant traits in variable environments. Funct. Plant Biol. 36, 665–681. doi: 10.1071/FP09144 32688679

[B15] Dong N. Lin H. (2021). Contribution of phenylpropanoid metabolism to plant development and plant–environment interactions. J. Integr. Plant Biol. 63, 180–209. doi: 10.1111/jipb.13054 33325112

[B16] Evans D. R. Guy H. I. (2004). Mammalian pyrimidine biosynthesis: fresh insights into an ancient pathway. J. Biol. Chem. 279, 33035–33038. doi: 10.1074/jbc.R400007200 15096496

[B17] Figueroa C. M. Lunn J. E. (2016). A tale of two sugars: trehalose 6-phosphate and sucrose. Plant Physiol. 172, 7–27. doi: 10.1104/pp.16.00417 27482078 PMC5074632

[B18] Garssen A. G. Baattrup-Pedersen A. Voesenek L. A. C. J. Verhoeven J. T. A. Soons M. B. (2015). Riparian plant community responses to increased flooding: a meta-analysis. Glob. Change Biol. 21, 2881–2890. doi: 10.1111/gcb.12921 25752818

[B19] Gibbs J. Greenway H. (2003). Review: mechanisms of anoxia tolerance in plants. I. Growth, survival and anaerobic catabolism. Funct. Plant Biol. 30, 353. doi: 10.1071/PP98095_ER 32689018

[B20] Grubbs S. A. Taylor J. M. (2004). The influence of flow impoundment and river regulation on the distribution of riverine macroinvertebrates at Mammoth Cave National Park, Kentucky, U.S.A. Hydrobiologia 520, 19–28. doi: 10.1023/B:HYDR.0000027722.23374.dc

[B21] Hirabayashi Y. Mahendran R. Koirala S. Konoshima L. Yamazaki D. Watanabe S. . (2013). Global flood risk under climate change. Nat. Clim. Change 3, 816–821. doi: 10.1038/nclimate1911 37880705

[B22] Huang C.-H. Chen W.-C. Gao Y.-H. Hsiao H.-I. Pan C.-L. (2021). Production of phenyllactic acid from Porphyra residues by lactic acid bacterial fermentation. Processes 9, 678. doi: 10.3390/pr9040678 30654563

[B23] IPCC (2014). Climate change 2014: Synthesis report (Geneva, Switzerland: IPCC).

[B24] Jeong H. Tombor B. Albert R. Oltvai Z. N. Barabasi A.-L. (2000). The large-scale organization of metabolic networks. Nature 407, 651–654. doi: 10.1038/35036627 11034217

[B25] Jones M. E. (1980). Pyrimidine nucleotide biosynthesis in animals: genes, enzymes, and regulation of UMP biosynthesis. Annu. Rev. Biochem. 49, 253–279. doi: 10.1146/annurev.bi.49.070180.001345 6105839

[B26] Keddy P. A. (2010). Wetland ecology: Principles and conservation (Cambridge: Cambridge University Press). doi: 10.1017/CBO9780511778179

[B27] Kleczkowski L. A. Geisler M. Ciereszko I. Johansson H. (2004). UDP-glucose pyrophosphorylase: an old protein with new tricks. Plant Physiol. 134, 912–918. doi: 10.1104/pp.103.036053 15020755 PMC523891

[B28] Koch K. E. (1996). Carbohydrate-modulated gene expression in plants. Annu. Rev. Plant Physiol. Plant Mol. Biol. 47, 509–540. doi: 10.1146/annurev.arplant.47.1.509 15012299

[B29] Konnerup D. Pedersen O. (2017). Flood tolerance of Glyceria fluitans: the importance of cuticle hydrophobicity, permeability and leaf gas films for underwater gas exchange. Ann. Bot. 120, 521–528. doi: 10.1093/aob/mcx083 29059317 PMC5737359

[B30] Konnerup D. Winkel A. Herzog M. Pedersen O. (2017). Leaf gas film retention during submergence of 14 cultivars of wheat (Triticum aestivum). Funct. Plant Biol. 44, 877–887. doi: 10.1071/FP16401 32480616

[B31] Kurokawa Y. Nagai K. Huan P. D. Shimazaki K. Qu H. Mori Y. . (2018). Rice leaf hydrophobicity and gas films are conferred by a wax synthesis gene (LGF1) and contribute to flood tolerance. New Phytol. 218, 1558–1569. doi: 10.1111/nph.15070 29498045

[B32] Lei S. Zeng B. Yuan Z. Su X. (2014). Changes in carbohydrate content and membrane stability of two ecotypes of Calamagrostis arundinacea growing at different elevations in the drawdown zone of the Three Gorges Reservoir. PloS One 9, e91394. doi: 10.1371/journal.pone.0091394 24608821 PMC3946822

[B33] Lindow S. E. Brandl M. T. (2003). Microbiology of the phyllosphere. Appl. Environ. Microbiol. 69, 1875–1883. doi: 10.1128/AEM.69.4.1875-1883.2003 12676659 PMC154815

[B34] Löffler M. Fairbanks L. D. Zameitat E. Marinaki A. M. Simmonds H. A. (2005). Pyrimidine pathways in health and disease. Trends Mol. Med. 11, 430–437. doi: 10.1016/j.molmed.2005.07.003 16098809

[B35] Macfarlane W. W. Gilbert J. T. Jensen M. L. Gilbert J. D. Hough-Snee N. McHugh P. A. . (2017). Riparian vegetation as an indicator of riparian condition: Detecting departures from historic condition across the North American West. J. Environ. Manage. 202, 447–460. doi: 10.1016/j.jenvman.2016.10.054 27839846

[B36] Mommer L. Visser E. J. W. (2005). Underwater photosynthesis in flooded terrestrial plants: A matter of leaf plasticity. Ann. Bot. 96, 581–589. doi: 10.1093/aob/mci212 16024559 PMC4247027

[B37] Mori Y. Kurokawa Y. Koike M. Malik A. I. Colmer T. D. Ashikari M. . (2019). Diel O_2_ dynamics in partially and completely submerged deepwater rice: Leaf gas films enhance internodal O_2_ status, influence gene expression and accelerate stem elongation for “snorkelling” during submergence. Plant Cell Physiol. 60, 973–985. doi: 10.1093/pcp/pcz009 30668838

[B38] Nosonovsky M. Bhushan B. (2005). Roughness optimization for biomimetic superhydrophobic surfaces. Microsyst. Technol. 11, 535–542. doi: 10.1007/s00542-005-0602-9 17570591

[B39] Nosonovsky M. Bhushan B. (2007). Hierarchical roughness optimization for biomimetic superhydrophobic surfaces. Ultramicroscopy 107, 969–979. doi: 10.1016/j.ultramic.2007.04.011 17570591

[B40] Nurrahma A. H. I. Yabuta S. Junaedi A. Sakagami J.-I. (2021). Analysis of non-structural carbohydrate in relation with shoot elongation of rice under complete submergence. Sustainability 13, 670. doi: 10.3390/su13020670 30654563

[B41] Overdieck D. Ziche D. Yu R. (2013). Gas exchange of Populus euphratica leaves in a riparian zone. J. Arid. Land 5, 531–547. doi: 10.1007/s40333-013-0178-7 30311153

[B42] Pathoumthong P. Zhang Z. Roy S. Habti A. E. (2023). Rapid non -destructive method to phenotype stomatal traits. Plant Methods 19, 36. doi: 10.1186/s13007-023-01016-y 37004073 PMC10064510

[B43] Pedersen O. Rich S. M. Colmer T. D. (2009). Surviving floods: Leaf gas films improve O_2_ and CO_2_ exchange, root aeration, and growth of completely submerged rice. Plant J. 58, 147–156. doi: 10.1111/j.1365-313X.2008.03769.x 19077169

[B44] Plaxton W. C. Podestá F. E. (2006). The functional organization and control of plant respiration. Crit. Rev. Plant Sci. 25, 159–198. doi: 10.1080/07352680600563876 37339054

[B45] Ponzio A. Rebecchi A. Zivoli R. Morelli L. (2024). Reuterin, phenyllactic acid, and exopolysaccharides as main antifungal molecules produced by lactic acid bacteria: A scoping review. Foods 13, 752. doi: 10.3390/foods13050752 38472865 PMC10930965

[B46] Pramanik M. Shelley I. Adhikary D. Islam M. (2016). Carbohydrate reserve and aerenchyma formation enhance submergence tolerance in rice. Progressive Agric. 27, 256–264. doi: 10.3329/pa.v27i3.30805 40208441

[B47] Riederer M. Schreiber L. (2001). Protecting against water loss: Analysis of the barrier properties of plant cuticles. J. Exp. Bot. 52, 2023–2032. doi: 10.1093/jexbot/52.363.2023 11559738

[B48] Riis T. Kelly-Quinn M. Aguiar F. C. Manolaki P. Bruno D. Bejarano M. D. . (2020). Global overview of ecosystem services provided by riparian vegetation. BioScience 70, 501–514. doi: 10.1093/biosci/biaa041

[B49] Sand-Jensen K. Krause-Jensen D. (1997). Broad-scale comparison of photosynthesis in terrestrial and aquatic plant communities. Oikos 80, 203–208. doi: 10.2307/3546536

[B50] Sato Y. Ashihara H. (2008). Pyrimidine salvage and catabolism in leaves of mangrove species. Plant Sci. 174, 140–148. doi: 10.1016/j.plantsci.2007.10.005 38826717

[B51] Singh S. Pathak N. Fatima E. Negi A. S. (2021). Plant isoquinoline alkaloids: advances in the chemistry and biology of berberine. Eur. J. Med. Chem. 226, 113839. doi: 10.1016/j.ejmech.2021.113839 34536668

[B52] Skinner O. S. Blanco-Fernández J. Goodman R. P. Kawakami A. Shen H. Kemény L. V. . (2023). Salvage of ribose from uridine or RNA supports glycolysis in nutrient-limited conditions. Nat. Metab. 5, 765–776. doi: 10.1038/s42255-023-00774-2 37198474 PMC10229423

[B53] Stasolla C. Katahira R. Thorpe T. A. Ashihara H. (2003). Purine and pyrimidine nucleotide metabolism in higher plants. J. Plant Physiol. 160, 1271–1295. doi: 10.1078/0176-1617-00929 14658380

[B54] Su X. Bejarano M. D. Yi X. Lin F. Ayi Q. Zeng B. (2020). Unnatural flooding alters the functional diversity of riparian vegetation of the Three Gorges Reservoir. Freshw. Biol. 65, 1585–1595. doi: 10.1111/fwb.13523 40046247

[B55] Thorpe W. H. Crisp D. J. (1946). Studies on plastron respiration: I. The biology of Aphelocheirus (Hemiptera, Aphelocheiridae) and the mechanism of plastron retention. J. Exp. Biol. 24, 227–269. doi: 10.1242/jeb.24.3-4.227 18920741

[B56] Thorpe W. H. Crisp D. J. (1947a). Studies on plastron respiration: II. The respiratory efficiency of the plastron in Aphelocheirus. J. Exp. Biol. 24, 270–303. doi: 10.1242/jeb.24.3-4.270 18920742

[B57] Thorpe W. H. Crisp D. J. (1947b). Studies on plastron respiration: III. The orientation responses of Aphelocheirus (Hemiptera, Aphelocheiridae) in relation to plastron respiration; together with an account of specialized pressure receptors in aquatic insects. J. Exp. Biol. 24, 310–328. doi: 10.1242/jeb.24.3-4.310 18920744

[B58] Thorpe W. H. Crisp D. J. (1949). Studies on plastron respiration: IV. Plastron respiration in the Coleoptera. J. Exp. Biol. 26, 219–260. doi: 10.1242/jeb.26.3.219 15395898

[B59] Traut T. W. (1994). Physiological concentrations of purines and pyrimidines. Mol. Cell. Biochem. 140, 1–22. doi: 10.1007/BF00928361 7877593

[B60] Urbanič G. Politti E. Rodríguez-González P. M. Payne C. Schook D. M. Alves P. . (2022). Riparian zones—From policy neglected to policy integrated. Front. Environ. Sci. 10, 868527. doi: 10.3389/fenvs.2022.868527

[B61] Vedel F. Lalanne É. Sabar M. Chétrit P. De Paepe R. (1999). The mitochondrial respiratory chain and ATP synthase complexes: composition, structure and mutational studies. Plant Physiol. Biochem. 37, 629–643. doi: 10.1016/s0981-9428(00)80093-5

[B62] Verboven P. Pedersen O. Ho Q. T. Nicolai B. M. Colmer T. D. (2014). The mechanism of improved aeration due to gas films on leaves of submerged rice. Plant Cell Environ. 37, 2433–2452. doi: 10.1111/pce.12300 24548021

[B63] Voesenek L. A. C. J. Bailey-Serres J. (2015). Flood adaptive traits and processes: an overview. New Phytol. 206, 57–73. doi: 10.1111/nph.13209 25580769

[B64] Vorholt J. A. (2012). Microbial life in the phyllosphere. Nat. Rev. Microbiol. 10, 828–840. doi: 10.1038/nrmicro2910 23154261

[B65] Weyers J. D. B. Johansen L. G. (1985). Accurate estimation of stomatal aperture from silicone rubber impressions. New Phytol. 101, 109–115. doi: 10.1111/j.1469-8137.1985.tb02820.x 33873818

[B66] Winkel A. Colmer T. D. Ismail A. M. Pedersen O. (2013). Internal aeration of paddy field rice (Oryza sativa) during complete submergence – importance of light and floodwater O_2_. New Phytol. 197, 1193–1203. doi: 10.1111/nph.12048 23215967

[B67] Winkel A. Colmer T. D. Pedersen O. (2011). Leaf gas films of Spartina anglica enhance rhizome and root oxygen during tidal submergence. Plant Cell Environ. 34, 2083–2092. doi: 10.1111/j.1365-3040.2011.02405.x 21819414

[B68] Winkel A. Herzog M. Konnerup D. Floytrup A. H. Pedersen O. (2017). Flood tolerance of wheat – the importance of leaf gas films during complete submergence. Funct. Plant Biol. 44, 888–898. doi: 10.1071/FP16395 32480617

[B69] Winkel A. Pedersen O. Ella E. Ismail A. M. Colmer T. D. (2014). Gas film retention and underwater photosynthesis during field submergence of four contrasting rice genotypes. J. Exp. Bot. 65, 3225–3233. doi: 10.1093/jxb/eru166 24759881 PMC4071835

[B70] Winkel A. Visser E. J. W. Colmer T. D. Brodersen K. P. Voesenek L. A. C. J. Sand-Jensen K. . (2016). Leaf gas films, underwater photosynthesis and plant species distributions in a flood gradient. Plant Cell Environ. 39, 1537–1548. doi: 10.1111/pce.12717 26846194

[B71] Wu Z. Liang X. Li M. Ma M. Zheng Q. Li D. . (2023). Advances in the optimization of central carbon metabolism in metabolic engineering. Microb. Cell Fact. 22, 76. doi: 10.1186/s12934-023-02090-6 37085866 PMC10122336

[B72] Xu H. Li C. Wang M. Guo Y. Zhang S. Ge Y. (2024). Enhancement of disease resistance against Alternaria alternata in winter jujube fruit by phenyllactic acid through regulating Ca^2+^ signaling transduction and mitogen-activated protein kinase cascades. Postharvest Biol. Technol. 210, 112774. doi: 10.1016/j.postharvbio.2024.112774 38826717

[B73] Yan Y. Li X. Zhang C. Lv N. Gao Y. Li H. (2021). Research progress on antibacterial activities and mechanisms of natural alkaloids: a review. Antibiotics 10, 318. doi: 10.3390/antibiotics10030318 33808601 PMC8003525

[B74] Yeung E. van Veen H. Vashisht D. Paiva A. L. S. Hummel M. Rankenberg T. . (2018). A stress recovery signaling network for enhanced flooding tolerance in Arabidopsis thaliana. Proc. Natl. Acad. Sci. U.S.A. 115, E6085–E6094. doi: 10.1073/pnas.1803841115 29891679 PMC6042063

[B75] Zhang Z. L. (2003). Experimental guide for plant physiology (Beijing: Higher Education Press).

[B76] Zrenner R. Stitt M. Sonnewald U. Boldt R. (2006). Pyrimidine and purine biosynthesis and degradation in plants. Annu. Rev. Plant Biol. 57, 805–836. doi: 10.1146/annurev.arplant.57.032905.105421 16669783

