# Supplementary material for: Leaf gas films enhance metabolic responses to submergence in Cynodon dactylon
Source: Front Plant Sci. 2026 Jun 2;17:1786650. doi: 10.3389/fpls.2026.1786650 (PMC13269078; doi:10.3389/fpls.2026.1786650)
Supplement: Supplementary Table 1 — KEGG pathway descriptions and column chart compositions. [file Table1.docx]

Supplementary Materials and Methods S1

Targeted metabolomics experimental methods

**1** **Experimental Methods**

**1.1 Metabolites Extraction**

(1) Put 20 mg of sample into a 2 mL centrifuge tube, add steel balls, and add 1 mL acetonitrile: water solution (7:3, V/V, containing standard mixture) to vortex for 30s;

(2) 25 Hz homogenate for 10 min, then vortex for 3min, place on ice and extract for 30 min;

(3) The sample was centrifuged at 4 ° C at 12000 r/min for 10 min;

(4) Take the supernatant 500 μ L over 0.22 μ M membrane injection analysis.

**1.2 Standard Solution Preparation**

Accurately transfer the corresponding amount of standard (1000mg/L) into a 10 mL volumetric flask to prepare a 10 mg/L mixed standard solution. A series of calibration solutions that dilute this standard solution in turn.

**1.3 UHPLC-MRM-MS Analysis**

The UHPLC separation was carried out using an Waters ACQUITY I-Class, equipped with a ACQUITY UPLC BEH Amide 1.7μm （1.7μm 2.1*150mm，Waters）. The mobile phase A2: Water: acetonitrile =95:5 (containing 0.05% ammonia and 5µM methylene diphosphate); B1: Water: acetonitrile =5:95 (containing 0.05% ammonia and 5µM methylene diphosphate). The column temperature was set at 40°C. The auto-sampler temperature was set at 10°C and the injection volume was 1 μL.

A SCIEX QTRAP 6500+ triple quadrupole mass spectrometer (Sciex), equipped with an IonDrive Turbo V electrospray ionization (ESI) interface, was applied for assay development. Typical ion source parameters were: Curtain Gas = 20 psi, IonSpray Voltage = +5500 V, -4500V, temperature = 500°C, Ion Source Gas 1 = 50 psi, Ion Source Gas 2 = 55 psi.

The MRM parameters for each of the targeted analytes were optimized using flow injection analysis, by injecting the standard solutions of the individual analytes, into the API source of the mass spectrometer. Several most sensitive transitions were used in the MRM scan mode to optimize the collision energy for each Q1/Q3 pair. Among the optimized MRM transitions per analyte, the Q1/Q3 pairs that showed the highest sensitivity and selectivity were selected as ‘quantifier’ for quantitative monitoring. The additional transitions acted as ‘qualifier’ for the purpose of verifying the identity of the target analytes.

SCIEX Analyst Work Station Software (Version 1.7.2) and Sciex OS 2.0.1 were employed for MRM data acquisition and processing.

**1.4 Calibration Curves**

Calibration solutions were subjected to UPLC-MRM-MS/MS analysis using the methods described above. Least squares method was used for the regression fitting. 1/x weighting was applied in the curve fitting since it provided highest accuracy and correlation coefficient (R). The level was excluded from the calibration if the accuracy of calibration was not within 80%–120%.

**1.5 Limit of Detection (LOD) and Limit of Quantitation (LOQ)**

The calibration standard solution was diluted stepwise, with a dilution factor of 2. These standard solutions were subjected to UHPLC-MRM-MS analysis. The signal-to-noise ratios (S/N) were used to determine the lower limits of detection (LLODs) and lower limits of quantitation (LLOQs). The LLODs and LLOQs were defined as the analyte concentrations that led to peaks with signal-to-noise ratios (S/N) of 3 and 10, respectively, according to the US FDA guideline for bioanalytical method validation.

**1.6 Precision and Accuracy**

The precision of the quantitation was measured as the relative standard deviation (RSD), determined by injecting analytical replicates of a QC sample. The accuracy of quantitation was measured as the analytical recovery of the QC sample determined. The percent recovery was calculated as [(mean observed concentration) / (spiked concentration)] × 100%.

The names of 57 metabolites: L-Arginine, Beta-Leucine, L-Lysine, L-Ornithine, L-Threonine, L-Citrulline, L-Glutamic acid, L-Asparagine, L-Serine, L-Alanine, L-Tyrosine, L-Glutamine, Phosphorylethanolamine, Guanosine, c-di-AMP, Adenine, L-Cystine, Acetyl-CoA, Succinyl-CoA, Succinic acid, Citric-acid, Fumaric acid, Alpha-Ketoglutaric acid, Oxaloacetate, Isocitric acid, ATP, Guanosine diphosphate, ADP, cyclic-AMP, Uracil, dAMP, dTMP, AMP, dCMP, IMP, UMP, dUMP, UDP-GlcNAc, D-Ribulose 5-phosphate, Flavin Mononucleotide, 6-Phosphogluconic acid, Phenyllactate, Glycerol 3-phosphate, Dihydroxyacetone phosphate, D-Glucose 6-phosphate, L-Lactate, 2-Phospho-D-glyceric acid, Phosphoenolpyruvic acid, Pyruvic acid, Fructose 1,6-bisphosphate, Inosine, Malic Acid, Itaconic Acid, cis-Aconitic acid, D-Fructose 6-phosphoric acid, Dl-Glyceric Acid, Glyoxalic Acid
