# Supplementary figures and images for: Leaf gas films enhance metabolic responses to submergence in Cynodon dactylon
Source: Front Plant Sci. 2026 Jun 2;17:1786650. doi: 10.3389/fpls.2026.1786650 (PMC13269078; doi:10.3389/fpls.2026.1786650)

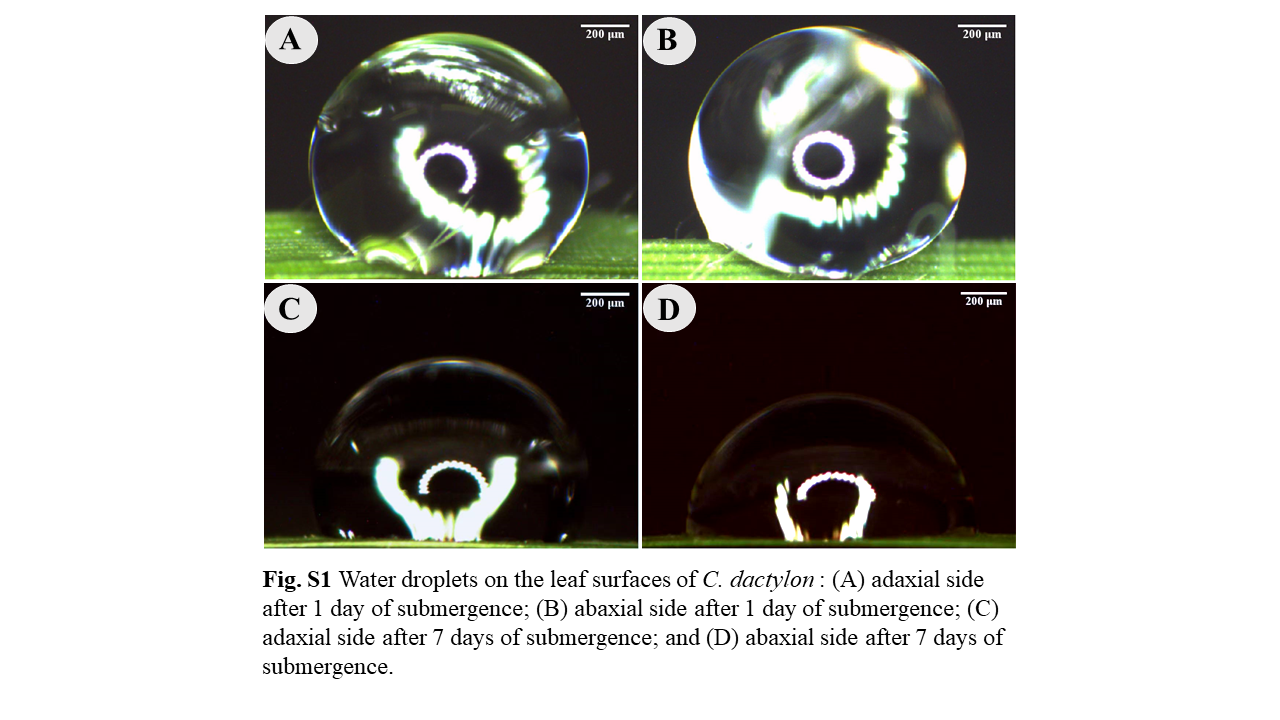

Supplement: Supplementary Figure 1 — Water droplets on the leaf surfaces of C. dactylon. [file Image1.tif]

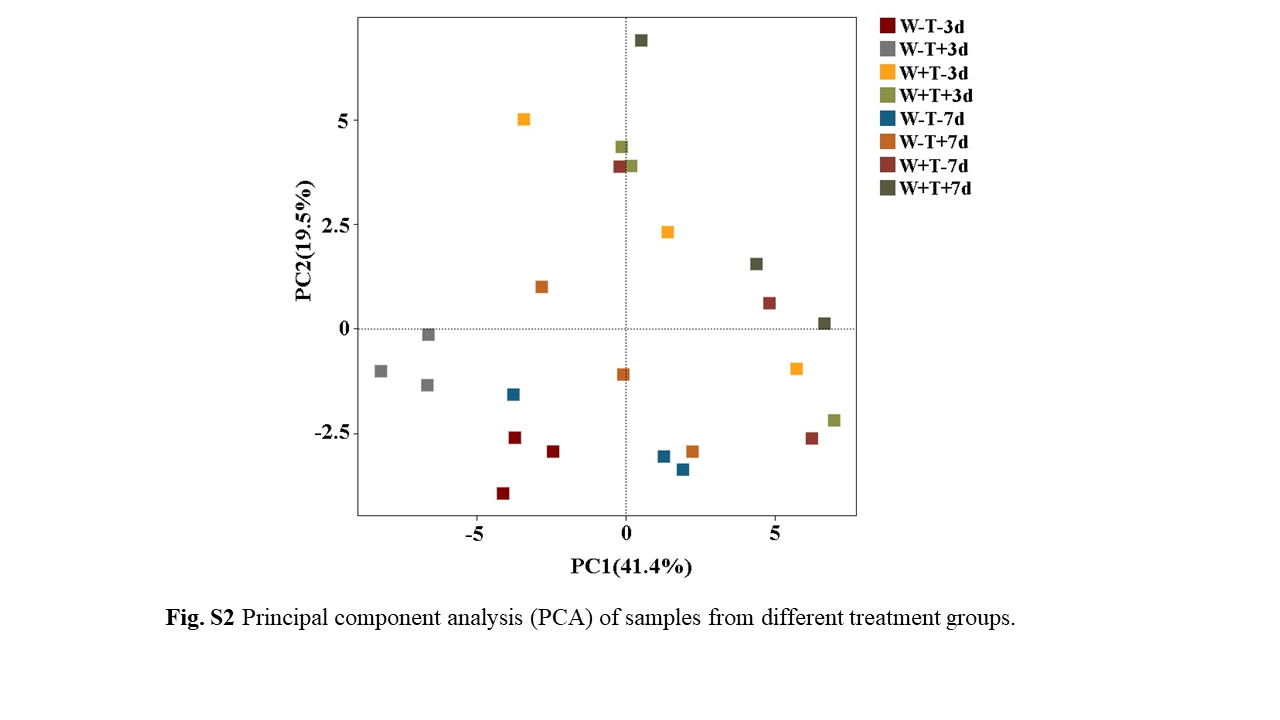

Supplement: Supplementary Figure 2 — Principal component analysis (PCA) of samples. [file Image2.tif]

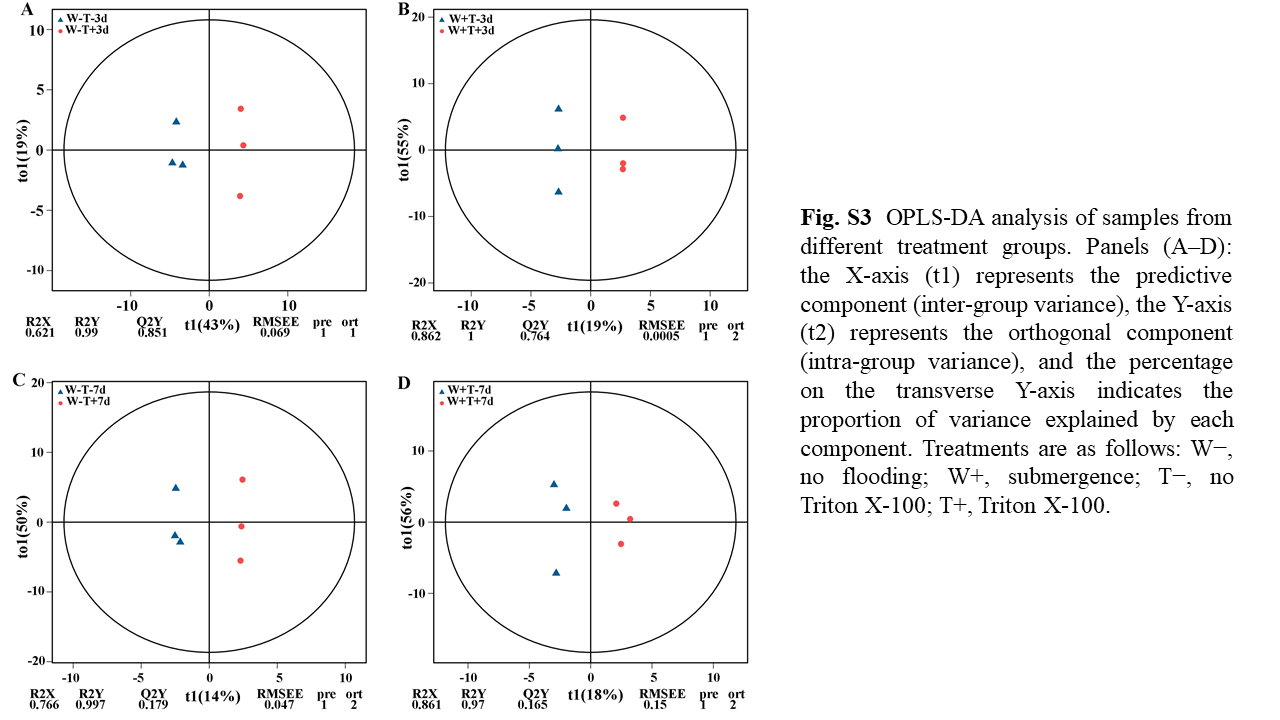

Supplement: Supplementary Figure 3 — OPLS-DA analysis of samples. [file Image3.tif]

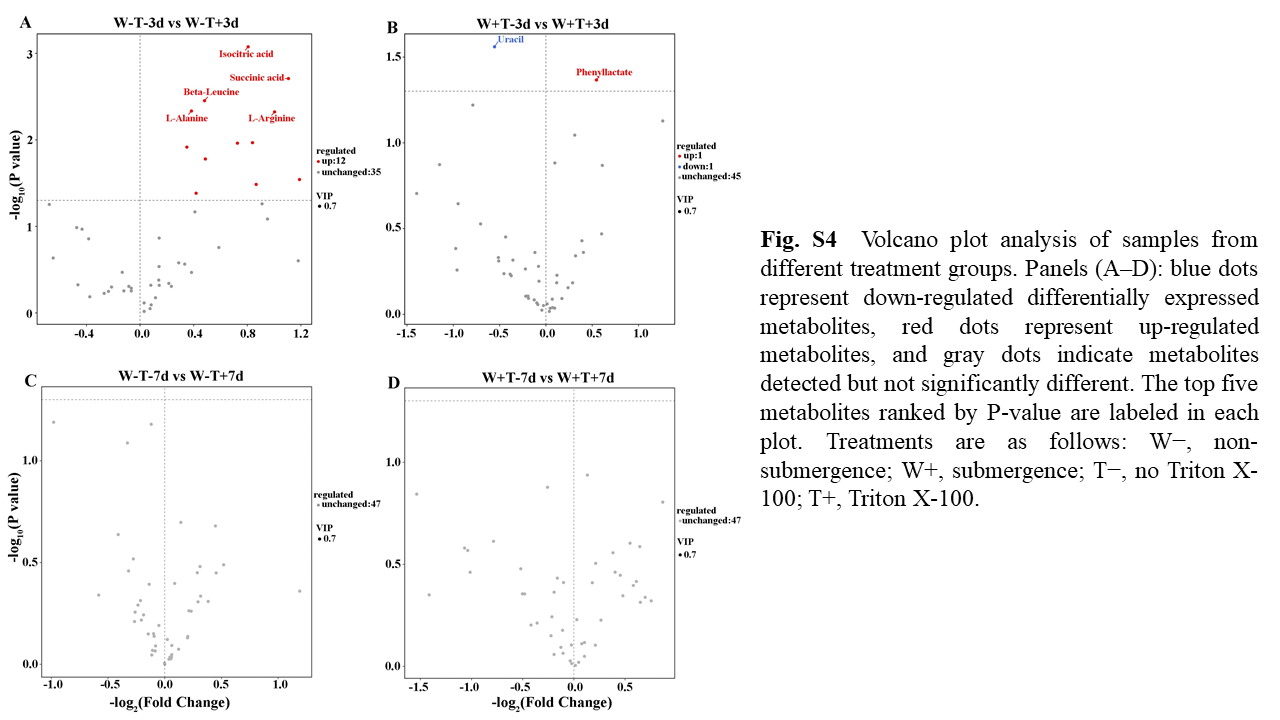

Supplement: Supplementary Figure 4 — Volcano plot analysis of samples. [file Image4.tif]

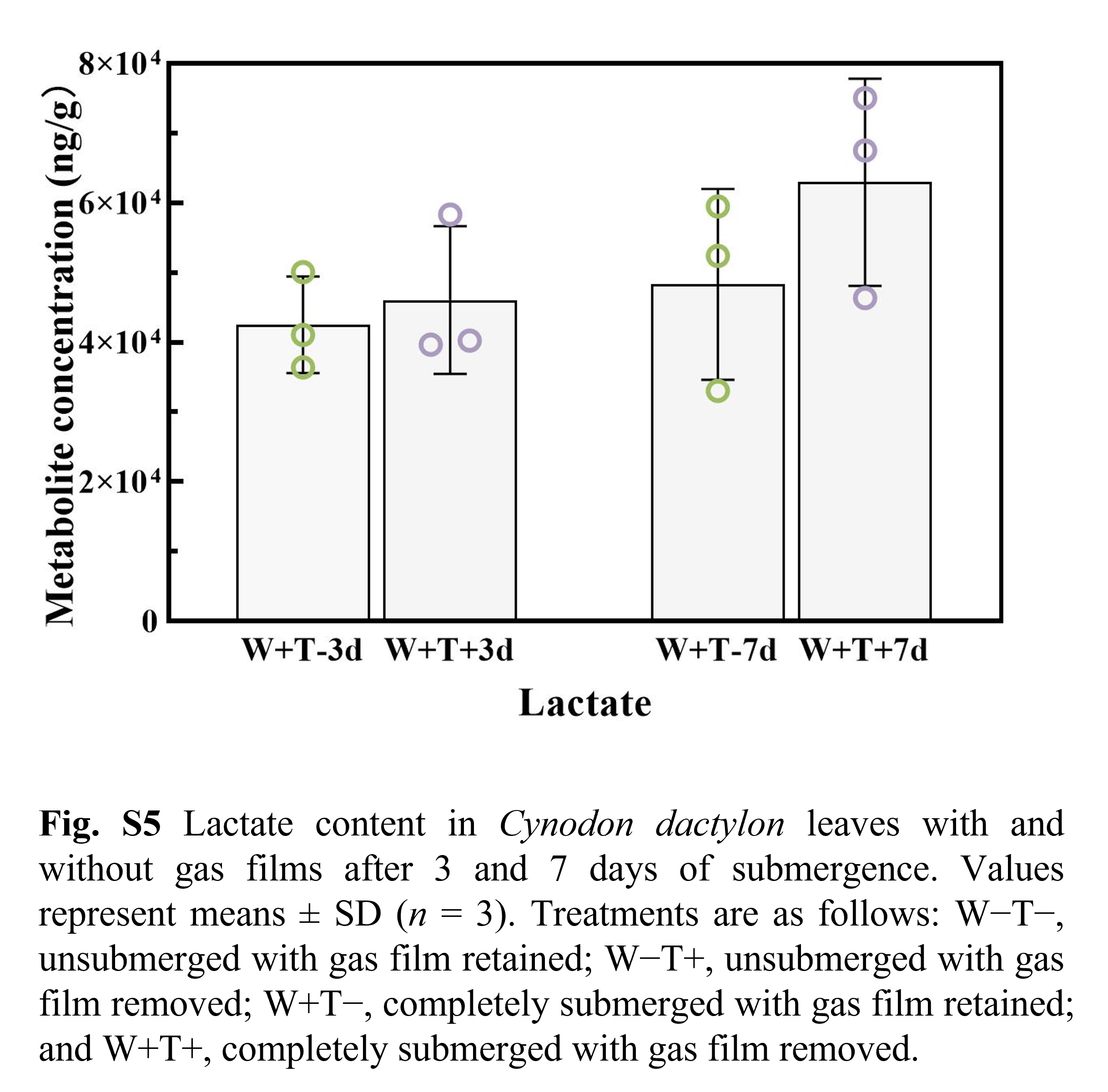

Supplement: Supplementary Figure 5 — Lactate content at 3 and 7 days of submergence. [file Image5.tif]
